# Mitochondrial RNA modification-based signature to predict prognosis of lower grade glioma: a multi-omics exploration and verification study

**DOI:** 10.1038/s41598-024-63592-w

**Published:** 2024-06-01

**Authors:** Xingwang Zhou, Yuanguo Ling, Junshuan Cui, Xiang Wang, Niya Long, Wei Teng, Jian Liu, Xin Xiang, Hua Yang, Liangzhao Chu

**Affiliations:** 1 Department of Neurosurgery, The Affiliated Hospital of Guizhou Medical University, Guiyang, Guizhou Province People’s Republic of China; 2 Department of Neurosurgery, Guizhou Provincial People’s Hospital, Guiyang, Guizhou Province People’s Republic of China

**Keywords:** Lower grade glioma, Mitochondrial RNA modification, Epigenetic modification, Single-cell analysis, Bulk RNA-seq analysis, CNS cancer, CNS cancer, Data mining

## Abstract

Mitochondrial RNA modification (MRM) plays a crucial role in regulating the expression of key mitochondrial genes and promoting tumor metastasis. Despite its significance, comprehensive studies on MRM in lower grade gliomas (LGGs) remain unknown. Single-cell RNA-seq data (GSE89567) was used to evaluate the distribution functional status, and correlation of MRM-related genes in different cell types of LGG microenvironment. We developed an MRM scoring system by selecting potential MRM-related genes using LASSO regression analysis and the Random Survival Forest algorithm, based on multiple bulk RNA-seq datasets from TCGA, CGGA, GSE16011, and E-MTAB-3892. Analysis was performed on prognostic and immunological features, signaling pathways, metabolism, somatic mutations and copy number variations (CNVs), treatment responses, and forecasting of potential small-molecule agents. A total of 35 MRM-related genes were selected from the literature. Differential expression analysis of 1120 normal brain tissues and 529 LGGs revealed that 22 and 10 genes were upregulated and downregulated, respectively. Most genes were associated with prognosis of LGG. METLL8, METLL2A, TRMT112, and METTL2B were extensively expressed in all cell types and different cell cycle of each cell type. Almost all cell types had clusters related to mitochondrial RNA processing, ribosome biogenesis, or oxidative phosphorylation. Cell–cell communication and Pearson correlation analyses indicated that MRM may promoting the development of microenvironment beneficial to malignant progression via modulating NCMA signaling pathway and ICP expression. A total of 11 and 9 MRM-related genes were observed by LASSO and the RSF algorithm, respectively, and finally 6 MRM-related genes were used to establish MRM scoring system (TRMT2B, TRMT11, METTL6, METTL8, TRMT6, and TRUB2). The six MRM-related genes were then validated by qPCR in glioma and normal tissues. MRM score can predict the malignant clinical characteristics, abundance of immune infiltration, gene variation, clinical outcome, the enrichment of signaling pathways and metabolism. In vitro experiments demonstrated that silencing METTL8 significantly curbs glioma cell proliferation and enhances apoptosis. Patients with a high MRM score showed a better response to immunotherapies and small-molecule agents such as arachidonyl trifluoromethyl ketone, MS.275, AH.6809, tacrolimus, and TTNPB. These novel insights into the biological impacts of MRM within the glioma microenvironment underscore its potential as a target for developing precise therapies, including immunotherapeutic approaches.

## Introduction

Glioma is a prevalent tumor in the central nervous system (CNS), representing 28% of all brain tumors and 80% of malignant tumors^1,2,3^. The World Health Organization (WHO) classification identifies grade II and III gliomas as types of Lower Grade Gliomas (LGGs), which have an extended survival time in patients compared to those with grade IV glioma^4^. Local recurrence or progression to glioblastoma is inevitable despite patients with LGGs being subjected to gross total resection, chemotherapy, and radiation treatment^2,5,6^. The significance of tumor molecular subtypes and prognostic biomarkers in the diagnosis and treatment of CNS tumors is underscored in the most recent edition of the WHO classification^6,7^. Classic molecules such as IDH1, TERT, and 1p19q have been employed for the routine clinical diagnosis and prognostic prediction of LGGs. However, these factors are inadequate to overcome the dilemma of glioma treatment and prognosis due to different genetic backgrounds. Therefore, it is crucial to identify novel biomarkers for individualized therapeutic targets and the prognostic prediction of LGGs.

Mitochondrial RNA modification (MRM) is an important way for regulating the expression of important mitochondrial genes. Modifications of mitochondrial RNAs are mainly used to regulate the stability, structure, and translation efficiency of mRNAs encoded by the mitochondrial genome. Accumulated evidence shows that different mitochondrial RNAs (e.g., rRNA, tRNA, or mRNA) can be extensively modified. Each modification is highly regulated and involves various regulatory proteins^8–11^. MRM is essential for maintaining cellular metabolic pathways and overall cell homeostasis, which, if disrupted, can lead to disease ^12^. Recent findings by Delaunay et al. indicate that MRMs play a significant role in producing mitochondrial proteins involved in glycolysis and oxidative phosphorylation (OXPHOS), potentially aiding the metastatic spread of cancer cells^13^. They stated that the control of mitochondrial mRNA translation to enhance metastasis is governed by 5-methylcytosine (m5C) and its derivative, 5-formylcytosine (f5C), which are dependent on NOP2/Sun RNA methyltransferase 3 (NSUN3)^14^. However, in-depth studies on MRM in LGGs have not been conducted.

This study leveraged single-cell RNA sequencing (scRNA-seq) data for the first time to explore the role of MRM within the LGG microenvironment, focusing on cellular communication, immune checkpoints (ICPs), and functional status. Moreover, machine learning techniques were utilized to identify potential MRM-related genes and develop an MRM-related signature. This signature was then analyzed concerning somatic mutations, copy number variations, prognosis, immune cell infiltration, and treatment responses, offering new insights into the molecular dynamics within LGGs.

## Materials and methods

### Datasets

Single-cell RNA-seq data (GSE89567) were extracted from Gene Expression Omnibus (GEO; https://www.ncbi.nlm.nih.gov/geo/query/acc.cgi?acc=gse89567). A total of 8 LGG patients with 6,341 cells were identified for single-cell analysis. RNA-seq data from various sources, including TCGA-LGG, CGGA1 (mRNAseq_693 + mRNAseq_325), CGGA2 (mRNA-array_301), E-MTAB-3892, GSE16011, and REMBRANDT, along with their clinical information, were obtained from the Cancer Genome Atlas (TCGA; https://portal.gdc.cancer.gov/), CGGA (http://www.cgga.org.cn/), GEO (https://www.ncbi.nlm.nih.gov/geo/), and ArrayExpress (https://www.ebi.ac.uk/arrayexpress/). The GTEx dataset available at the UCSC website (http://xena.ucsc.edu/) was used to extract gene expression information of normal brain tissues^15^.

### Single-cell RNA-seq analysis

We used the “Seurat” R package to perform single-cell RNA-seq analysis^16^. First, the “FindVariableFeatures” algorithm was used to identify 2,000 highly differentially expressed genes (DEGs), and dimension reduction was performed based on these normalized genes. The “findneighbors” and “findclusters” algorithms were employed for the clustering and definition of cell subtypes, respectively, based on the top 20 PCs. The classic marker genes selected from the literature were used to define the cell subpopulations^16,17^. The “CellCycleScoring” function was used to calculate the cell cycle score. The “CellChat” R package was used to perform an analysis of the cellular communication network. Cell–cell communication network analysis led to the identification of strong signaling pathways in the LGG microenvironment. We conducted Pearson correlation analysis to assess the potential implications of MRM-related genes in modulating these signaling pathway networks and ICPs.

### Single-sample gene set enrichment analysis

To identify both the MRM-related signature and the 16 gene signatures mentioned above, the “GSVA” R package was utilized to carry out single-sample gene set enrichment analysis (ssGSEA)^18^. The MRM-related genes were obtained from previous research^8^. The ssGSEA was used to quantify immune cell infiltration based on bulk RNA-seq data.

### Identification of MRM-related signature genes

A Pearson correlation analysis was conducted to evaluate how the MRM-associated signature relates to gene expression in various cell types, including astrocytes, oligodendrocytes, endothelial cells, M1 and M2 macrophages, and glioma cells. The Cytoscape software, along with its Molecular Complex Detection (MCODE) plugin, was utilized to pinpoint core gene modules that had a score of over 10^19^.

### Bulk RNA-seq analysis

A predictive model was built to assess the expression and clinical prediction of MRM-related genes in LGGs in a comprehensive and systematic manner. We assessed the prognostic significance and differential expression of genes related to MRM^20^. The construction of the predictive model involving potential MRM-related genes was performed by utilizing LASSO regression analysis and the random Forest SRC software package for gene selection^21,22^. The Random Survival Forest (RSF) algorithm was applied to rank the importance of MRM-related genes^23^. The intersect MRM-related genes obtained via LASSO and the random forest algorithm were employed to establish the risk model through multivariate regression. The risk model was as follows: MRM score = TRMT2B(exp)*1.05 + TRMT11(exp)*(− 0.69) + METTL6(exp)*(− 1.13) + METTL8(exp)*1.30 + TRMT6(exp)*0.78 + TRUB2(exp)*(− 0.99). The prognostic role of the MRM score was validated for CGGA1, CGGA2, GSE16011, E-MTAB-3892, and REMBRANDT. The predictability of the MRM score was assessed through ROC analysis using the R software package timeROC, and compared to other variables like tumor grade, age, IDH mutation, and 1p19q codeletion. In addition, survival models based on the MRM score were assessed based on the concordance index. To assess the link of risk models’ predictive accuracy with that of clinical characteristics, we examined how age, tumor grade, treatment strategy, IDH mutation, 1p19q codeletion, and MRM score act as single and multiple predictors, and created a nomogram incorporating age, tumor grade, IDH mutation, 1p19q codeletion, and MRM score as clinical features.

### Somatic mutation and copy number alteration analysis

Mutation and CNV data were sourced from cBioPortal (https://www.cbioportal.org/), a comprehensive web resource that provides access to a wide array of molecular datasets. For visualization of somatic mutations across different MRM score groups, the OncoPrint tool from the “complexheatmap” package in R was employed. This approach enabled the clear representation of genetic alterations in patients classified into high and low MRM score groups. Genes exhibiting an association with MRM scores and demonstrating a mutation rate exceeding 10% were specifically identified for detailed analysis.

### Analysis of immune infiltration

In the present study, TIMER, ssGSEA, and MCPCOUNTER were used to quantify the infiltration of immune cells^18,24–26^. An online web tool called TIMER 2.0 (http://timer.cistrome.org/) could comprehensively quantify immune cell infiltration. The “GSVA” R package was used to determine the presence of 23 immune cells through ssGSEA. The MCPCOUNTER algorithm yielded 10 immune cells in total. The application of the ESTIMATE algorithm enabled the estimation of the immune score, stromal score, and tumor purity^27^. Immunomodulators involved in antigen presentation and cell adhesion, co-inhibitors, co-stimulators, ligands, and receptors, were identified based on a previous study^28^.

### Drug response prediction

The assessment of T-cell dysfunction and exclusion was carried out using the TIDE online algorithm (http://tide.dfci.harvard.edu/)^29–31^. Subsequently, the relationship between the MRM score and immune checkpoint blockade (ICB) therapy response (including anti-PD1 and anti-CTLA4 therapy) was analyzed^29^. The eXtreme Sum algorithm was utilized to identify the top 5 drugs demonstrating high sensitivity in patients with high MRM scores. This algorithm is particularly effective for highlighting treatments that might be most beneficial based on specific genetic or molecular profiles^32^.

### Quantitative real time polymerase chain reaction (RT-qPCR)

We extracted all RNA from 8 gliomas and 4 normal tissues using a triazole solution (Takara, Japan) and followed the standard protocol for synthesis. Tsingke Biotechnology (Beijing, China) synthesized the primer pairs, which were provided in Table S1. The relative expression levels of mRNA were calculated by 2^−△△CT^ method following normalizing all samples to GAPDH.

### Cell transfection

Hanbio (Shanghai, China) provided us with the siRNA targeting negative control (NC) and GPR82. After reaching 50% confluence in a 6-well plate, U87 and U251 cells were transfected with 50 nmol of NC and METTL8 siRNA using 5L of Lipofectamine 2000 reagent for 12 h.

### Clonogenic assay

U87 and U251 cells were transfected and incubated for 48 h. When individual cells formed clones consisting of more than 50 cells, the culture was terminated by removing the culture medium and washing the cells with PBS. The cells were fixed by adding ethanol and incubating for 30 min. After fixation, the fixative was carefully removed. The cells were then washed once with PBS, and the supernatant was carefully discarded. Afterwards, the cells were treated with a 0.1% crystal violet solution and incubated for 15–20 min for staining. Following the staining process, cells were washed with PBS to remove any excess stain effectively. After the washing step, the cells were left to air-dry, ensuring the removal of any remaining liquid. Finally, the cells were ready to be photographed. The plate was gently inverted, and a transparent grid film was placed over it. Clones were subsequently counted either by direct visual inspection or under a microscope using low magnification. This counting procedure was carried out when the number of cells in a clone exceeded 50. Finally, the clone formation rate was identified by estimating the ratio of the number of clones to the number of seeded cells, multiplied by 100%.

### Western blot

U87 and U251 cells were transfected for 48 h. The plate was placed in an incubator and cultured for 48 h. Protein extraction was executed using potent RIPA lysis buffer, succeeded by centrifugation at 12,000 rpm for 20 min at 4 °C to procure the supernatant. The protein concentrations were determined employing the BCA assay. SDS-PAGE facilitated the separation of proteins under a constant voltage of 120 V for 90 min. Subsequently, the proteins were translocated onto a PVDF membrane featuring a 0.45 μm pore size via a wet transfer system operating at a constant current of 400 mA for 60 min. The PVDF membrane underwent blocking with 5% skim milk (in TBST, Tris-buffered saline with Tween 20) for 1 h at ambient temperature. Primary antibodies targeting rabbit anti-human METTL8(Helmholtz Center Munich, 1:100 dilution) and GAPDH (Cell Signaling Technology, 1:1000 dilution) were applied and subjected to incubation. Post-incubation, the membrane was washed with TBST for 5 min, this procedure was replicated three times. HRP-conjugated secondary antibodies (1:3000) were then introduced and incubated for 1 h at room temperature. Following this, the membrane was washed again with TBST for 5 min, a step repeated three times. An ECL luminescent reagent was administered, and the membrane was imaged using a gel documentation system in an environment shielded from light. Bands of interest were subsequently analyzed utilizing Image J software.

### Apoptosis detection

We used flow cytometry to detect apoptosis. After washing the U87 and U251 cells with PBS, trypsin digestion was performed to collect the cells. The cell density was adjusted to 2 × 10^5^ cells/well with 2 mL/well in a 6-well plate, and both NC and transfection groups were established. The plate was incubated at 37 °C with 5% CO_2_. Once the cells adhered to the wells, cell transfection was performed. Depending on the grouping, the supernatant was collected in the corresponding numbered centrifuge tubes. Trypsin (EDTA) was added for digestion, and when the cells shrank and were no longer connected in sheets, the collected supernatant was added to stop the digestion and collected in the corresponding centrifuge tube. We resuspended U87 and U251 cells in 500 μL of Binding Buffer, and then 5 μL of Annexin V-APC was gently mixed in 5 μL of PI. The analysis was used flow cytometry after the reaction was carried out at room temperature in the dark for 15 min.

### Immunohistochemistry

Immunolabeling was conducted following a standard immunohistochemistry protocol^33^. Initially, samples were brought to room temperature, deparaffinized in xylene, and rehydrated through a series of graded ethanol washes. Heat-mediated antigen retrieval was carried out in a microwave using 10 mM sodium citrate buffer (pH 6.0) for 20 min. To prevent nonspecific background staining, a blocking solution containing 10% goat serum and 0.1% Triton X-100 in 1 × phosphate-buffered saline (PBS) was applied for 1 h at room temperature. Primary antibodies were then incubated overnight at 4 °C, followed by secondary antibodies for 1 h at room temperature. Double immunolabeling was performed in sequence, applying one set of primary and secondary antibodies followed by a second set. Cell nuclei were stained with DAPI (1 µg/ml; Invitrogen), and slides were mounted in Mowiol 4–88 mounting medium. The medium was prepared by mixing 2.4 g of Mowiol 4–88 with 6 g of glycerol, 6 mL of water, and 12 mL of 0.2 M Tris–Cl (pH 8.5), heating to 50 °C for 10 min, followed by centrifugation at 5000 g for 15 min. Finally, 2.5% DABCO was added to reduce fluorescence fading. We used the following primarily antibodies (diluted in 0.1% Triton X-100 in 1 × PBS) to perform immunohistochemistry: rabbit polyclonal antibody to GFAP (1:100; Agilent Technologies, Santa Clara, CA, USA), rabbit anti-human METTL8 (1:100 dilution, Helmholtz Center Munich).

### Statistical analysis

R software (version 4.1.0) was used to perform all statistical analyses. Student’s t-test was used to evaluate the disparities among the MRM score groups. The definition of statistical significance was that the value of p had to be less than 0.05.

## Results

### Extensive expression of METTL8, METTL2A, and METTL2B in the LGG microenvironment

**Figure 1:**
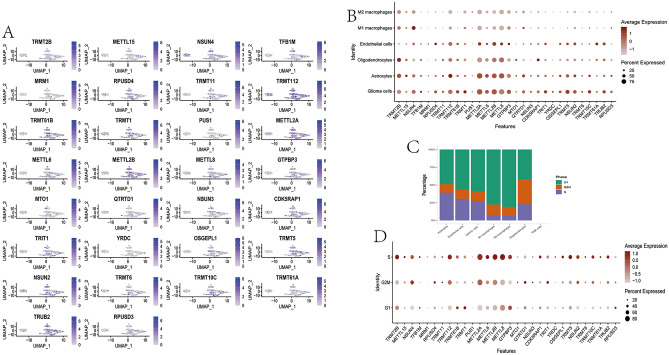
The distribution of MRM-related genes in LGG microenvironment. (**A**) A plot of MRM-related genes in 6 different cell types using tSNE. (**B**) The expression levels of MRM-related genes in 6 different cell types. (**C**) The distribution of cells across 3 different stages of the cell cycle in 6 different cell types. (**D**) The expression levels of MRM-related genes during 3 different stages of the cell cycle in 6 different cell types.

**Figure 2:**
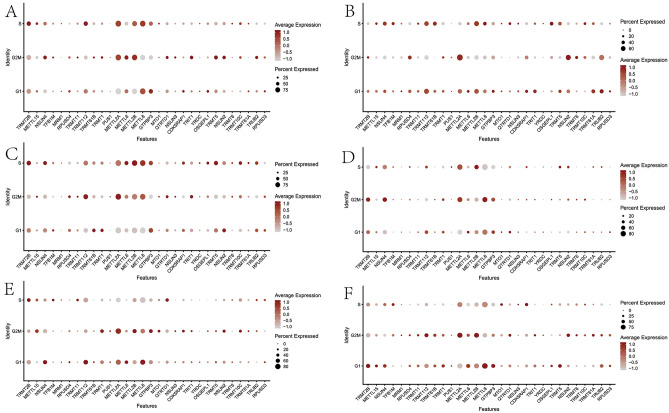
The presence of MRM-related genes in the three stages of the cell cycle for every cell type. (**A**) astrocyte; (**B**) endothelial cell; (**C**) glioma cell; (**D**) M1 macrophages; (**E**) M2 macrophages; (**F**) oligodendrocytes.

A total of 35 MRM-related genes were selected from the literature (Table S2). We firstly performed single-cell analysis to explore the expression of 35 MRM-related genes in LGG microenvironment based on a scRNA-seq dataset with 8 LGG patients. Specific cell types, including glioma cells (PTPRZ1), oligodendrocytes (SEPT4, CNP, PLP1, and MBP), astrocytes (ID3, GFAP, AQP4, and SOX9), M1 macrophages (CD68, CD74, TSPO, and CD86), M2 macrophages (CD68, CD74, and CD163), T cells or natural killer (NK) cells (CXCR4 and S100A4), and endothelial cells (A2M and APOLD1), were found to have a correspondence with high expression of gene sets. Later on, t-SNE plots were utilized to observe the expression of MRM-related genes in LGG samples (Fig. 1A). In general, MRM-related genes were expressed in 6 cell types at different degrees (Fig. 1A,B). METLL8, METLL2A, TRMT112, and METTL2B were extensively expressed in all cell types (Fig. 1B). GTPBP3 and TRMT2B were highly expressed in 5 cell types except for endothelial cells (Fig. 1B). Furthermore, we assessed the differential expression of MRM-related genes in cells with different cell cycle phases (Fig. 1C,D). We observed the high expression levels of METLL8, METLL2A, TRMT112, METTL2B, GTPBP3, METTL6, and NSUN4 in cells in the S, G2M, and G1 phases, which were similar for astrocytes, glioma cells, and oligodendrocytes (Fig. 2A,C,F). The MRM-related genes had different degrees of expression in M1 macrophages, M2 macrophages, and endothelial cells with different phases of cell cycle (Fig. 2B,D,F).

### Functional modules of MRM-related signature

Having demonstrated the MRM-related genes exhibited different expression patterns in various cells within the LGG microenvironment. We next identified genes associated with the MRM-related signature for each cell type by conducting Pearson correlation analysis. Using the Cytoscape software with MCODE (Max depth = 100, K-core = 2, Degree cutoff = 2, and Node score cutoff = 0.2), a PPI network was generated to display the functional modules of genes related to the MRM-associated signature. Enrichment analysis provided the possible BP and pathway for every cell type. Clusters associated with mitochondrial RNA processing, ribosome biogenesis, or oxidative phosphorylation were present in almost all cell types (Fig. S1 and Table S3).

### Signaling pathway networks associated with MRM-related genes

As we have determined that MRM-associated signature may be involved with biological process including mitochondrial RNA processing, ribosome biogenesis, or oxidative phosphorylation. We then analyzed cell–cell communication of all samples to investigate the strong signaling pathways in the LGG microenvironment and explored relationship between MRM-related genes and those signature pathways. We identified the NCAM (NCAM1 and NCAM2), PTN (PTN, PTPRZ1, NCL, SDC3, and SDC4), JAM (JAM2, JAM3, ITGAM, and ITGB2), PSAP (PSAP, GPR37L1, and GPR37), APP (APP and CD74), GALECTIN (LGALS9 and CD44), GRN (GRN and SORT1), NOTCH (DLL3 and NOTCH2), and MIF (CD74, MIF, and CXCR4) signaling pathway networks in majority of samples (Fig. S2A–I, Table S4). The JAM, APP, MIF, and NCAM signaling pathway networks were targeted by glioma cells, endothelial cells, astrocytes, and oligodendrocytes. Furthermore, other cells within the GRN and PSAP signaling pathways were regulated by glioma cells, M1 macrophages, M2 macrophages, and endothelial cells (Fig. S2C and E). We conducted Pearson correlation analysis to establish whether signaling pathway networks are regulated by MRM-related genes. In glioma cells, astrocytes, and oligodendrocytes, the genes in the NCAM signaling pathway network were found to be expressed at a high level. The positive correlation between NCMA1 and CDK5RAP1, METTL15, METTL2A, METTL6, NSUN2, NSUN3, QTRTD1, TRMT1, TRMT11, TRMT112, and TRMT61B was observed in glioma cells. The expression of NCMA1 in astrocytes showed positive correlation with METTL15, MRM1, NSUN4, RPUSD3, and TRMT11, while NCMA2 exhibited negative correlation with TFB1M, TRIT1, TRMT5, and YRDC (Fig. S3). Cell–cell communication and Pearson correlation analyses indicated that MRM may be involved in glioma progression via the NCMA signaling pathway by modulating astrocytes, which should be validated in further studies.

### Correlation of MRM-related genes with ICPs in the immune microenvironment

**Figure 3:**
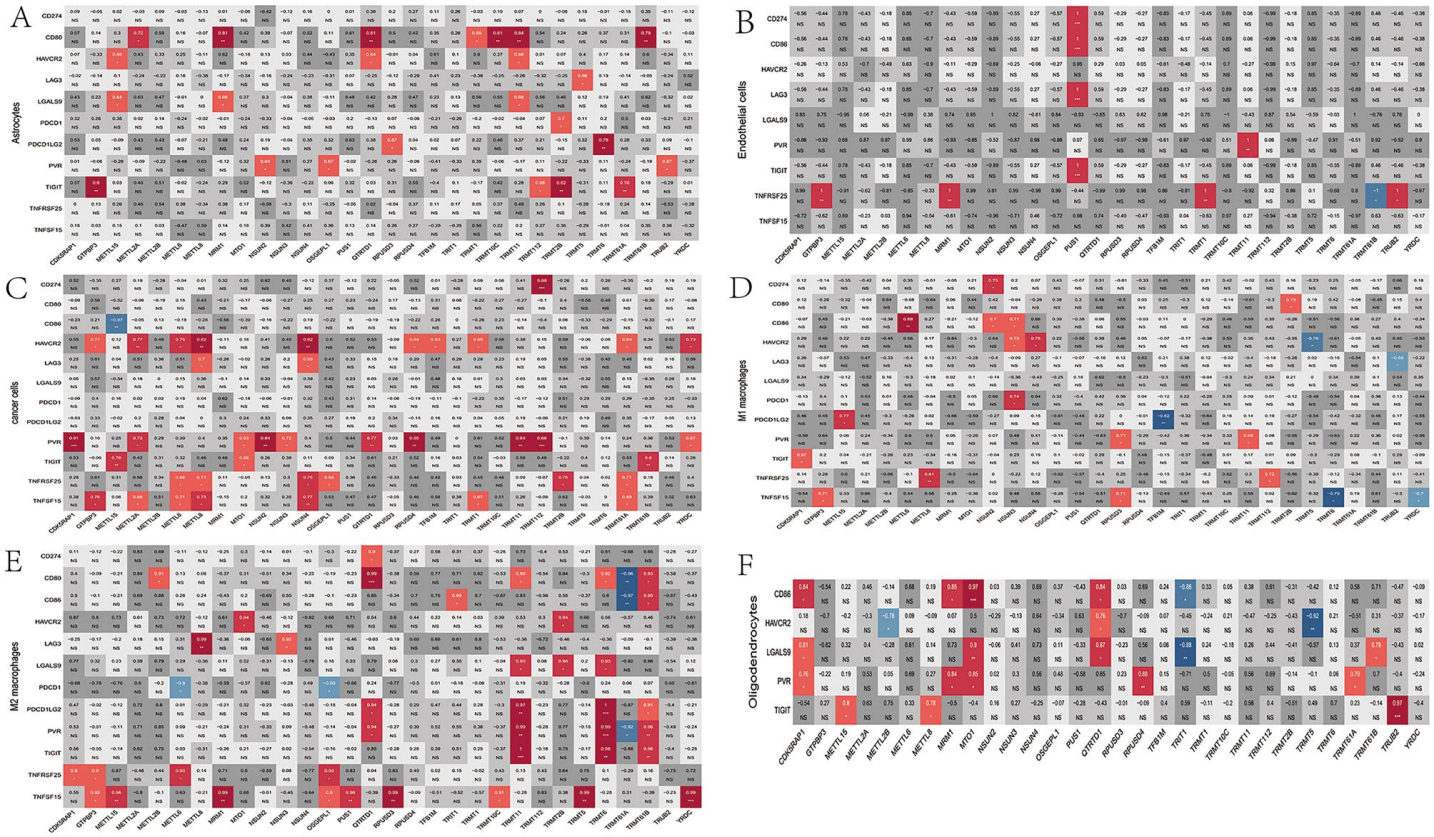
Correlation analysis of MRM-related genes with ICPs in 6 cell types. (**A**) astrocyte; (**B**) endothelial cell; (**C**) glioma cell; (**D**) M1 macrophages; (**E**) M2 macrophages; (**F**) oligodendrocytes.

In our findings, some cell clusters were linked to immune-related pathways like the immune response-regulating signaling pathway in M2 macrophages. Therefore, we investigated the correlation of MRM-related genes with ICPs in the immune microenvironment (Fig. 3). The expression of HAVCR2, PVR, TNFRSF25, and TNFSF15 showed a positive correlation with the expression of numerous MRM-related genes like GTPBP3, METTL2A, METTL6, METTL8, NSUN4, and TRMT61A in glioma cells. In M2 macrophages, TNFSF15 showed a positive correlation with GTPBP3, METTL15, MRM1, OSGEPL1, PUS1, RPUSD3, TRMT10C, TRMT5, and YRDC (Fig. 3B). In astrocytes, CD80 was positively correlated with METTL2A, MRM1, QTRTD1, TRMT1, TRMT10C, TRMT11, and TRMT61B (Fig. 3A). These results demonstrated that MRM may increase the expression of ICPs, promoting an immunosuppressive microenvironment.

### Landscape of MRM-related genes in LGGs

**Figure 4:**
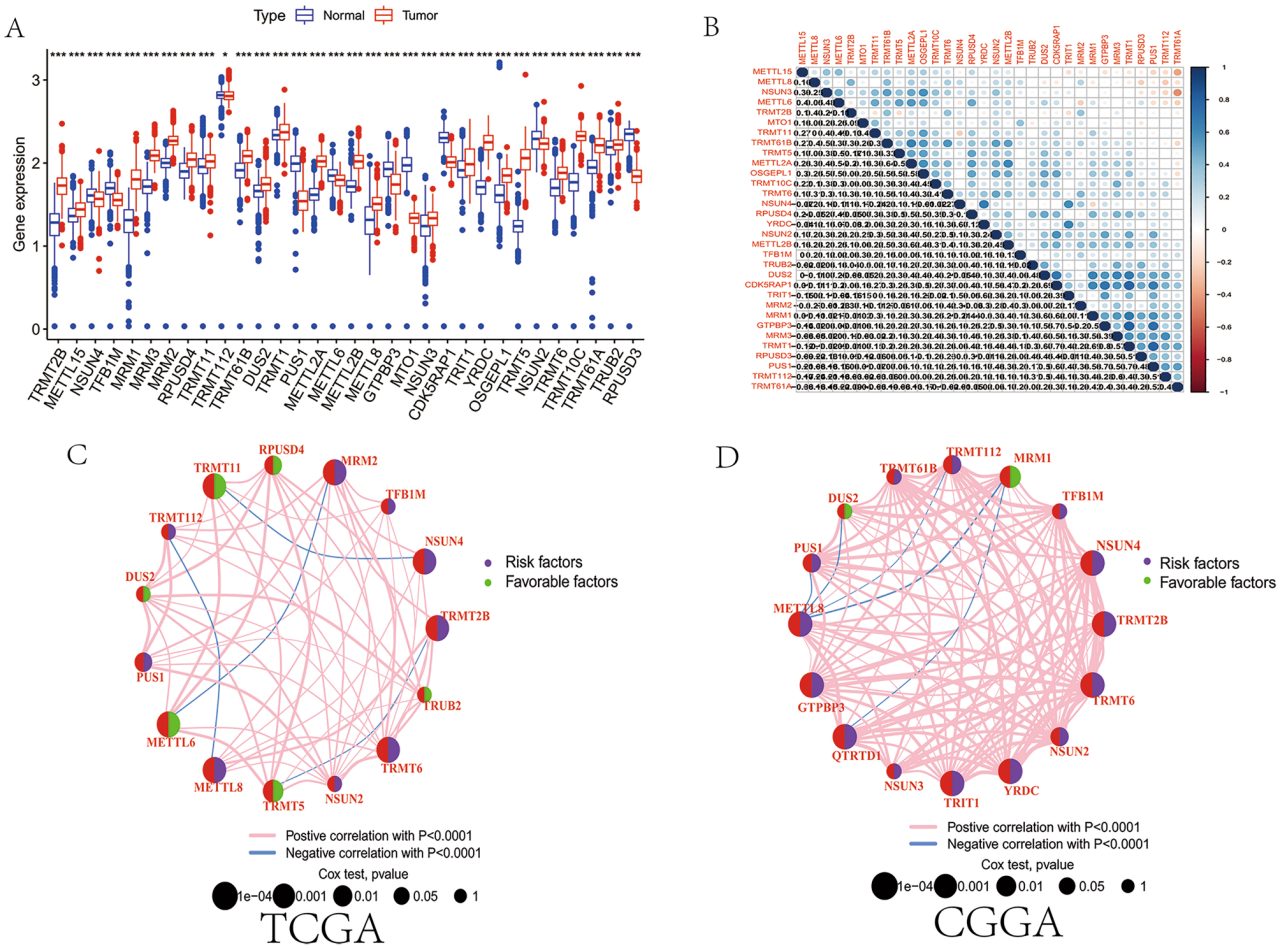
The expression and prognosis of MRM-related genes. (**A**) Gene expression of 32 MRM-related genes was examined in 1120 normal brain tissues and 529 LGG. (**B**) The correlation of MRM-related genes was studied. Survival and correlation analysis of MRM-related genes were conducted in TCGA (**C**) and CGGA1 (**D**) cohort, respectively. Purple solid dot represented that MRM-related gene was risk factor for LGG, while green represented that MRM-related gene was favorable factor. The orange line showed a positive correlation among MRM-related genes, but blue indicated negative correlation.

Following the single-cell data analysis mentioned above, we have established that genes related to MRM may play a significant role in the formation of a cancer-promoting microenvironment. We then assessed the expression patterns and prognostic role of those genes in LGG using several bulk RNA-seq datasets. Differential expression analysis of 1120 normal brain tissues and 529 LGGs revealed that 22 and 10 genes were upregulated and downregulated, respectively (Fig. 4A,B). We further evaluated the differential expression of these genes among different tumor grades and subtypes based on the IDH mutation status and 1p19q codeletion (subtype1: IDH mutant and 1p19q codeletion; subtype2: IDH mutant and 1p19q non-codeletion; IDH wild-type). TRMT2B, NSUN4, TFB1M, MRM3, MRM2, TRMT112, TRMT61B, TRMT1, PUS1, METTL8, GTPBP3, MTO1, YRDC, NSUN2, and TRMT6 were upregulated in grade III glioma, whereas METTL6 was downregulated in grade II glioma in the TCGA cohort (Fig. S4A). The differential expression of most MRM-related genes in different subtypes was statistically significant (Fig. S4B). Similar results were also obtained for the CGGA1 cohort (Fig. S4C and D). Analysis of the interaction patterns among the 35 MRM-related genes showed that TRMT5 was the hub node of MRM-related genes, followed by NSUN2, PUS1, TRMT61B, and its interactions with METLL2B, TRMT6B, and MRM1 were supported by the STRING database (Fig. S5A and B).

MRM-related genes’ prognostic value was determined through Cox proportional hazards regression analyses using a univariate model. A total of 15 genes (NSUN2, TRMT6, TURB2, TRMT2B, NSUN4, TFB1M, MRM2, RPUSD4, TRMT11, TRMT112, DUS2, PUS1, METTL6, METTL8, and TRMT5) were associated with the prognosis of patients with LGGs in the TCGA database; 9 genes were risk factors, whereas 6 genes were favorable factors (Fig. 4C). Similar results were also obtained by Cox proportional hazards regression analyses for the CGGA1 cohort (Fig. 4D).

Among the 508 patients with LGGs, the alteration frequency of 35 MRM-related genes was 1.78%, and TRTMT2B showed the highest mutation rate. Subsequently, we identified genes with a mutation rate greater than 10% associated with MRM-related genes. The high expression levels of CDK5RAP1, DUS2, METTL2A, METTL2B, METTL6, METTL15, MRM1, MRM3, TRIM1, TRTM5, TRTM11, TRMT61B, TRMT112, NSUN2, NSUN3, OSGEPL1, PUS1, RPUSD3, and RPUSD4 were associated with the high mutation rate of CIC, whereas the low expression levels of MRM2, TRIT1, YRDC, and NSUN4 were associated with the high mutation rate of CIC (Fig. S6A, B and C). The relationship between IDH1, TP53, and ATRX mutations and MRM-related genes is summarized in Fig. S6.

### Establishment of a predictive model based on MRM-related genes

**Figure 5:**
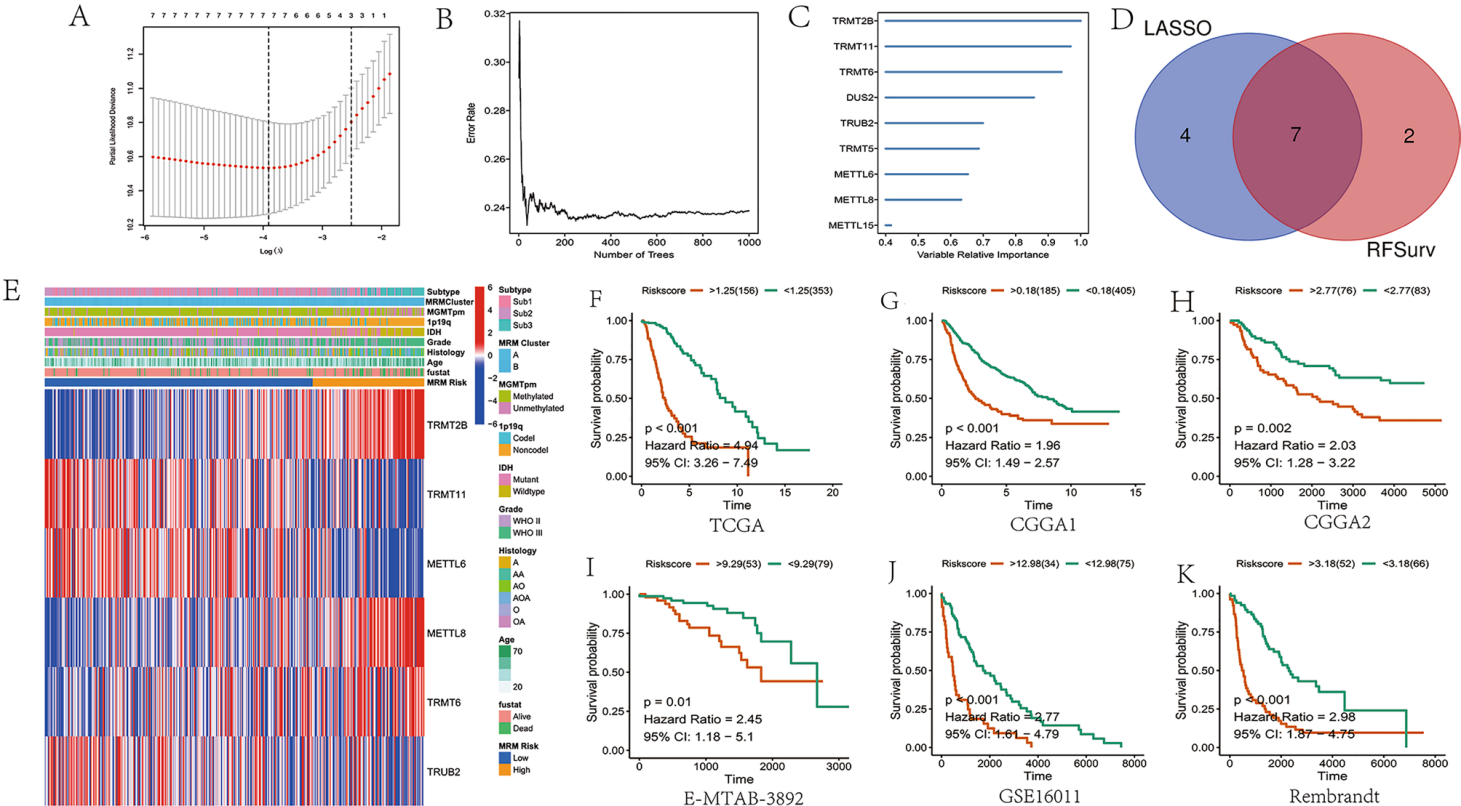
Risk model construction based on MRM-associated genes. (**A**) LASSO analysis; (**B** and **C**): RSF algorithms; (**D**) insect genes from LASSO analysis and RSF algorithms; (**E**) The distribution of clinical characteristics and 6 genes between low and high MRM score; The role of MRM score as a prognostic factor in LGG patients of TCGA (**F**), CGGA1 (**G**), CGGA2 (**H**), E-MATB-3892 (**I**), GSE16011 (**J**), Rembrandt (**K**) datasets, respectively.

**Figure 6:**
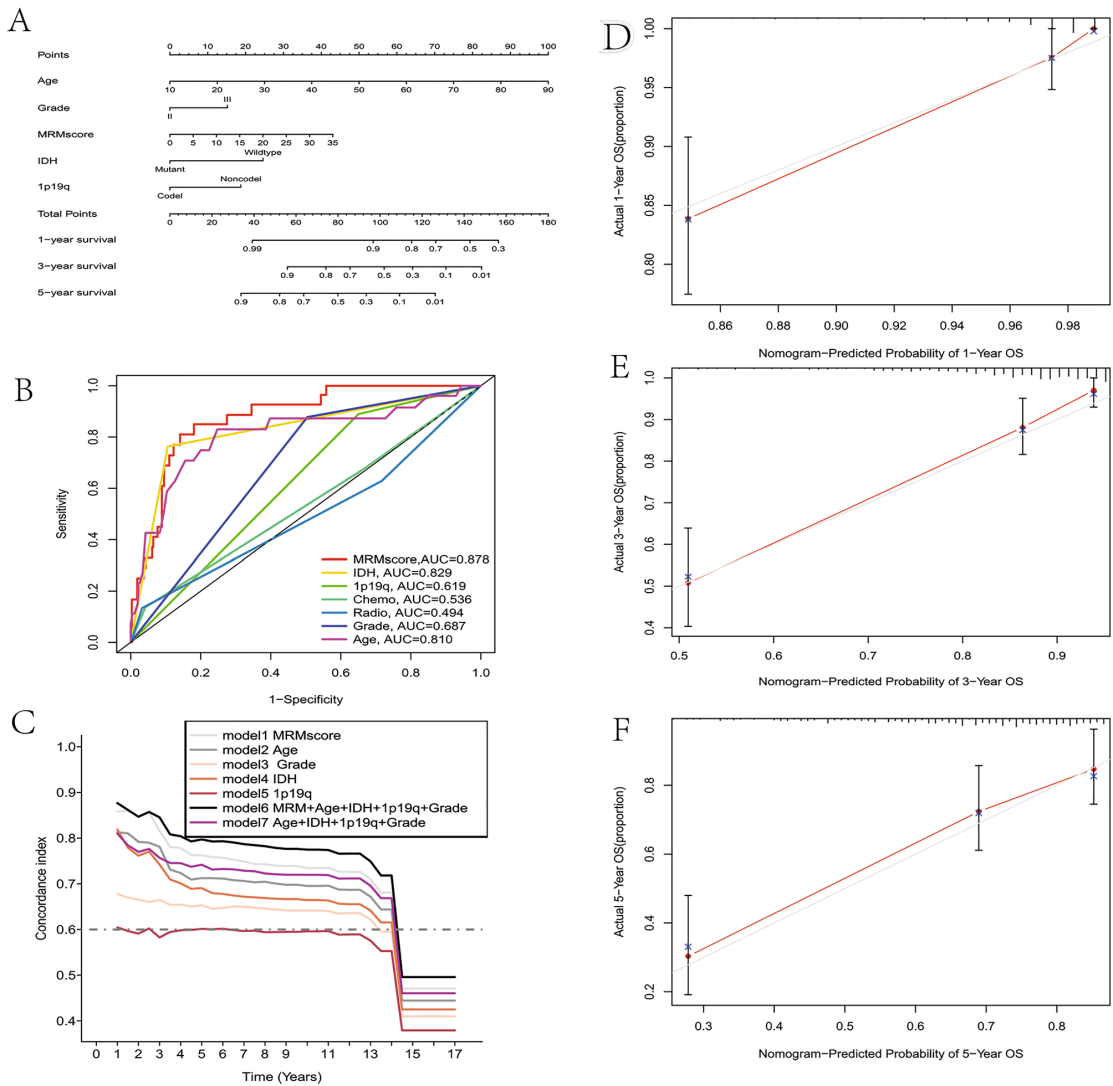
Nomogram predicting outcomes using risk model from TCGA database. (**A**) the nomogram based on the risk model; (**B**) The risk model displays a higher area under the curve (AUC) compared to age, IDH mutation status, 1p19q codeletion status, and tumor grade, as indicated by the ROC curve. (**C**) The concordance index of the model is based on MRM score and parameters. (**D**-**F**) The calibration curve for 1-year, 3-years and 5-years OS is created based on the nomogram, respectively.

**Figure 7:**
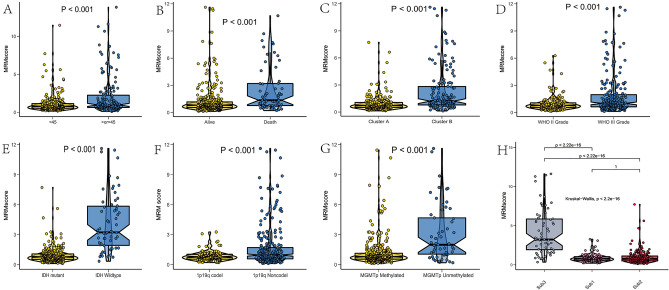
The relationship between MRM score and age (**A**), survival status(**B**), MRM cluster (**C**), tumor grade (**D**), IDH1 mutation (**E**), 1p19q codel (**F**), MGMT promoter methylation (**G**), subtypes (**H**), respectively.

Having demonstrated the expression patterns and prognostic role of MRM-related genes in LGG, we next used the RSF algorithm to perform LASSO regression analysis for choosing potential MRM-associated genes from bulk RNA-seq data to build a predictive model. The application of LASSO regression analysis and the RSF algorithm led to the identification of 11 and 9 MRM-related genes, respectively (Fig. 5A–C), and 6 intersect MRM-related genes were used to establish the predictive model (Fig. 5D). The TCGA database was utilized as the training set, while the CGGA1, CGGA2, gse16011, E-MTAB-3892, and REMBRANDT databases served as the testing sets (Fig. 5E). The predictive model was as follows: MRM score = TRMT2B(exp)*1.05 + TRMT11(exp)*(− 0.69) + METTL6(exp)*(− 1.30) + METTL8(exp)*1.13 + TRMT6(exp)*0.78 + TRUB2(exp)*(− 0.99). It was discovered that patients having a high MRM score had a shorter OS compared to those with a low MRM score. Additionally, the MRM score was concluded as an independent prognostic indicator for LGG patients in the TCGA cohort depicted in Fig. 5F. The CGGA1, CGGA2, gse16011, E-MTAB-3892, and REMBRANDT cohorts yielded comparable outcomes (Fig. 5G–K). A nomogram was used to visualize the final multivariable logistic regression model (Figs. S7 and 6A). The MRM score (AUC = 0.878) showed excellent predictive performance compared with that of IDH (AUC = 0.829), 1p19q (ACU = 0.619), radiation (AUC = 0.494), chemotherapy (AUC = 0.536), tumor grade (AUC = 0.687), and age (AUC = 0.810) (Fig. 6B). Furthermore, the concordance index of the model based on age, tumor grade, IDH mutation, 1p19q codeletion, and MRM score was higher than that of other models (Fig. 6C). Calibration curves were drawn, and the curve of the TCGA cohort was as expected (Fig. 6D,F). Similar results were obtained for the CGGA1 cohort (Fig. S8). Furthermore, we observed a correlation between elevated MRM score and malignancy features characterized by older age, mortality, absence of IDH1 mutation, higher tumor grading, lack of 1p19q co-deletion, and MGMT promoter methylation (Fig. 7).

DEGs between the high and low MRM score groups were identified (Fig. S9A and B). Analysis of the DEGs using GO revealed that the MRM score was linked to pathways related to the immune system, as demonstrated in Fig. S9C. The group with high MRM score exhibited enrichment of immune-related pathways, such as antigen processing and presentation as well as primary immunodeficiency, and leukocyte transendothelial migration, as indicated by GSVA analysis (Fig. S9D). We also systematically assessed the heterogeneity of tumor metabolism between the low and high MRM score groups. Our results showed that carbohydrate metabolism, lipid metabolism, nucleotide metabolism, and vitamin metabolism were abnormally active in the high MRM score group (Fig. S9E).

### Genomic mutation analysis based on the MRM score

**Figure 8:**
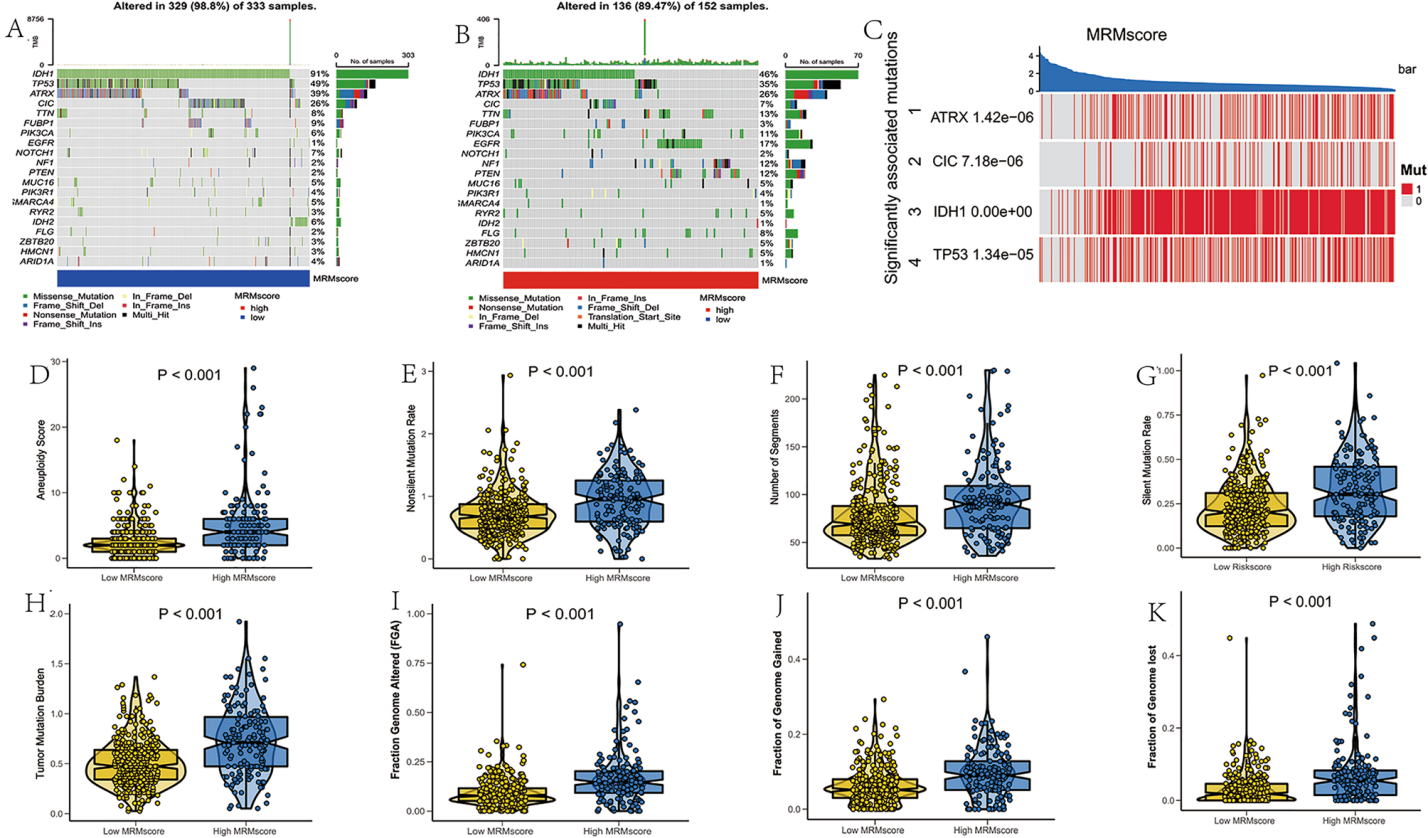
Genomic mutation analysis for MRM score. (**A**) genomic characterization landscape of low MRM score subgroup; (**B**) genomic characterization landscape of high MRM score subgroup; (**C**) genes with mutation rate greater than 10% associated with MRM score; (**D**) the association of MRM score with aneuploidy score, (**E**) nonsilent mutation rate, (**F**) number of segments, (**G**) silent mutation rate, (**H**) tumor mutation burden, (**I**) fraction genome altered, (**J**) fraction of genome gained and (**K**) fraction of genome lost.

**Figure 9:**
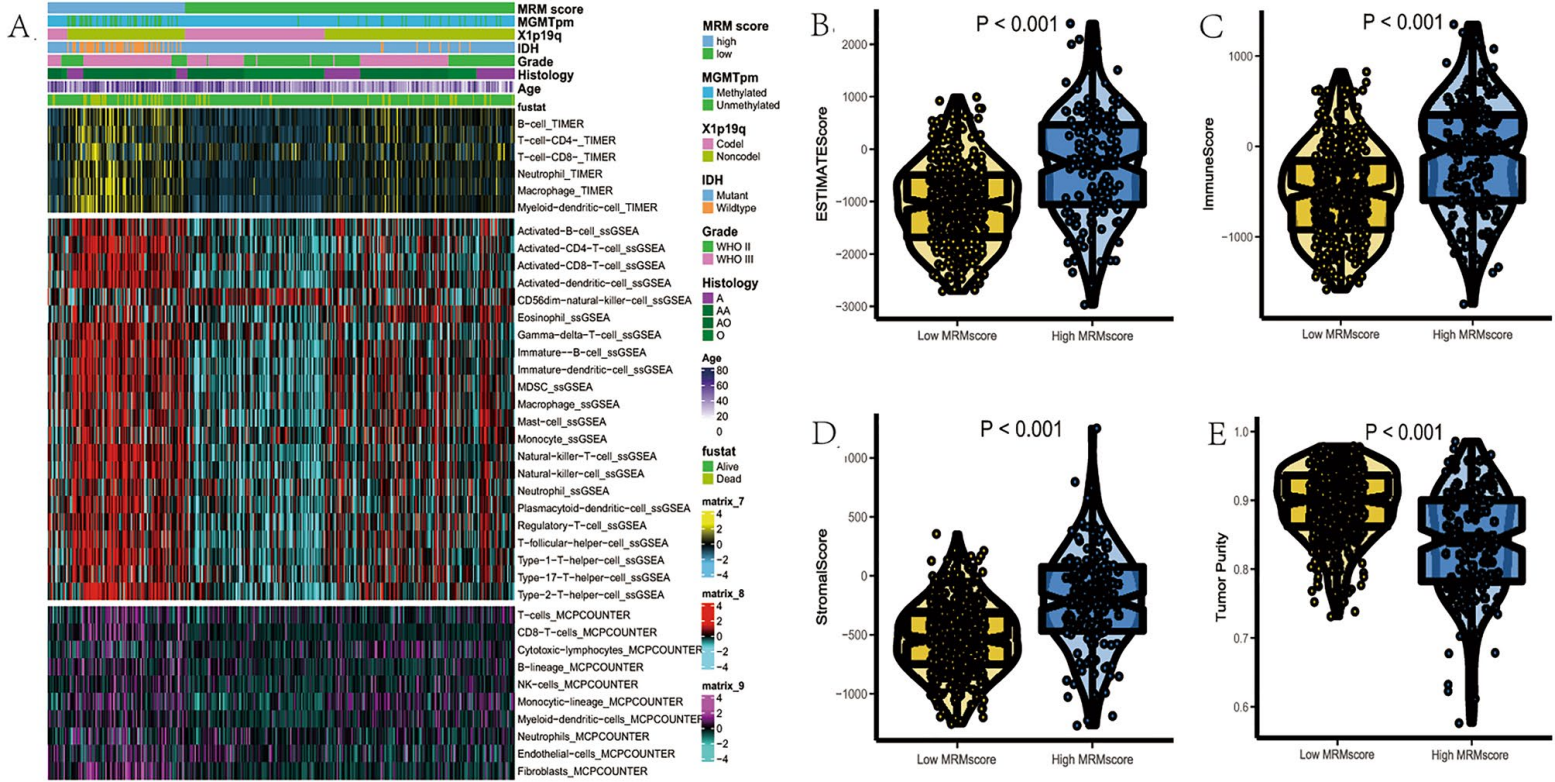
Immune infiltration for MRM signature in TCGA cohort. (**A**) infiltration of immune cells among MRM signature groups; distribution of ESTIMATEscore (**B**), immunescore (**C**), stromalscore (**D**), and tumor purity (**E**) among MRM subgroups.

As our results have shown, different MRM genes are associated with various common gene mutations, while the association between MRM score and genomic mutation. Therefore, an analysis was conducted on genomic mutation and SNV between groups with high and low MRM scores. Patients with low MRM score showed higher mutation rates of IDH1, TP53, ATRX, CIC, FUBP1, NOTCH1, SMARCA4, and IDH2 as compared to patients with high MRM score. Conversely, the mutation rates of TTN, EGFR, NF1, PTEN, and FLG were lower in patients with low MRM score (Fig. 9A,B). Furthermore, genes with a mutation rate greater than 10% associated with the MRM score were IDH1, CIC, TP53, and ATRX (Fig. 9C). Patients with low MRM score exhibited lower aneuploidy score, nonsilent mutation rate, number of segments, silent mutation rate, and tumor mutation burden compared to those with high MRM score (Fig. 8D–H). In addition, the high MRM score group had higher values for FGA, FGG, and FGL compared to the low MRM score group (Fig. 9I–K).

### Immune infiltration, immunotherapy and potential chemotherapy based on the MRM score.

**Figure 10:**
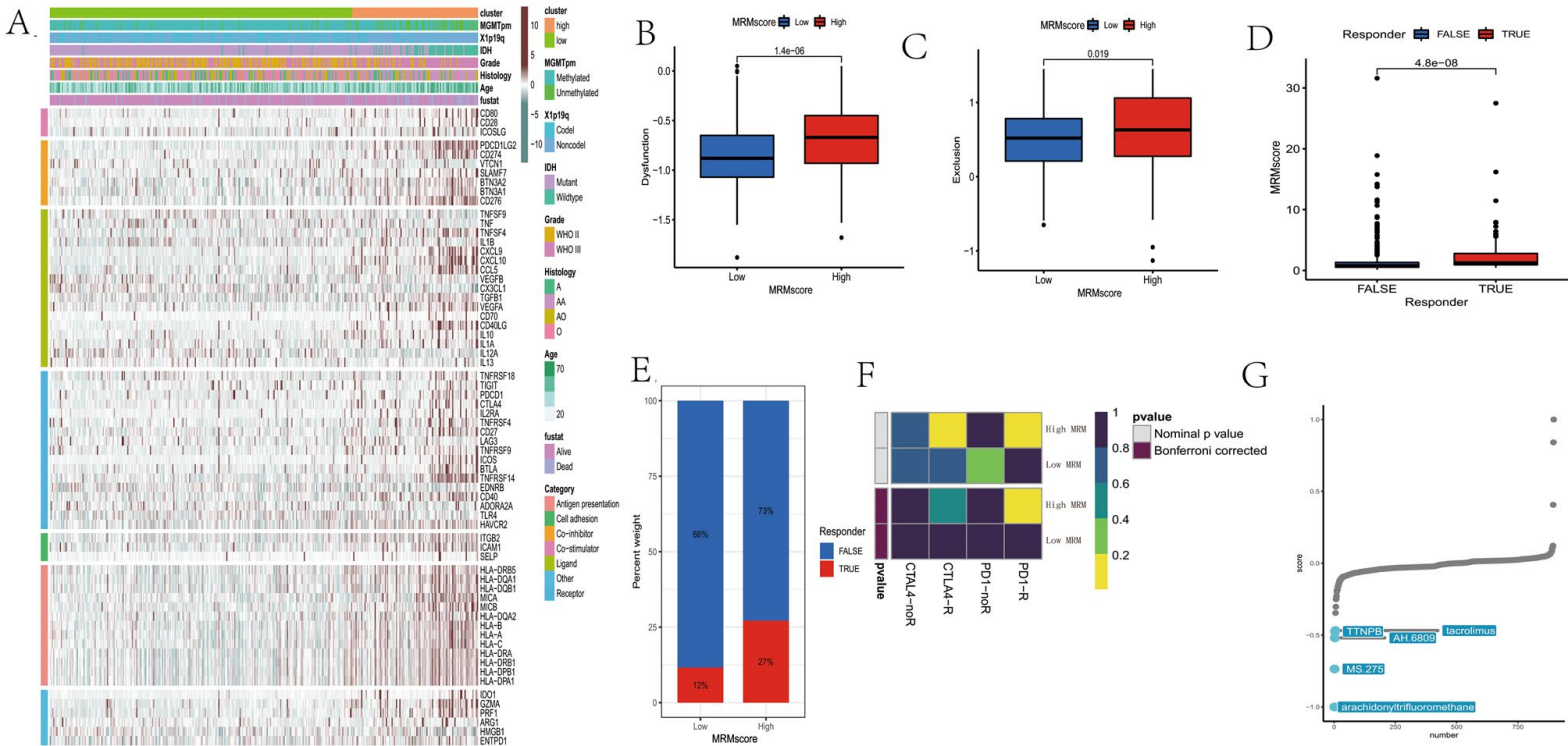
Immunotherapy and potential chemotherapy based on MRM score. (**A**) heatmap showed the association of MRM score with 7 immunomodulators in LGG; (**B**–**E**): the ability of MRM score in predicting immunotherapy response; (**F**) eXtreme Sum algorithm identified the top 5 sensitive drugs for high-MRM score group.

We have discovered that MRM genes were associated with immune related biological processes. Patients with high MRM score exhibited enrichment of antigen processing and presentation, primary immunodeficiency as well as leukocyte transendothelial migration. However, whether the MRM score can predict immune cell infiltration, immune therapy response, and screen for other potential treatment options remains unknown. The infiltration of immune cells was quantified in the current study using TIMER, ssGSEA, and MCPCOUNTER. The heatmap displays the prevalence of immune cells that are infiltrating for various MRM scores (Figs. 9A and S10). The group with high MRM score showed an increase in immune cell infiltration. Patients with high MRM scores exhibited elevated ESTIMATE, immune, and stromal scores, but decreased tumor purity relative to patients with low MRM scores (Fig. 9B–E). The heatmap indicated that ADORA2A, CX3CL1, EDNRB, HMGB1, IL12A, and VTCN1 had high expression levels in the low MRM score group. On the other hand, the high MRM score group displayed high expression levels of all immunomodulators except for VEGFB, TNFSF9, TNF, TLR4, TIGIT, SELP, LAG3, IL13, IL4, IL1B, IFNA2, and IFNA1 (Fig. 10A). The high expression of most immunomodulators in patients with high MRM score, as demonstrated by the TIDE algorithm, may provide reasoning for the potential benefit of immunotherapy in this group (Fig. 10B–E). In addition to TIDE prediction, we further perform subclass mapping to compare the expression profile of the two MRM subtypes and then assessed the response of immunotherapy. The result indicated that patients with high MRM score may benefit from anti–PD-1 therapy (Nominal *p* = 0.007, Bonferroni *p* = 0.056, Fig. 10F), even though the corrected *P* value did not reach statistical significance. Finally, we used the eXtreme Sum algorithm to identify sensitive drugs for patients with high MRM score. The most sensitive drug in the high MRM score group was arachidonyl trifluoromethyl ketone (AACOCF3), followed by MS.275, AH.6809, tacrolimus, and TTNPB (Fig. 10G).

### In vitro validation of the role of METTL8 in glioma

**Figure 11:**
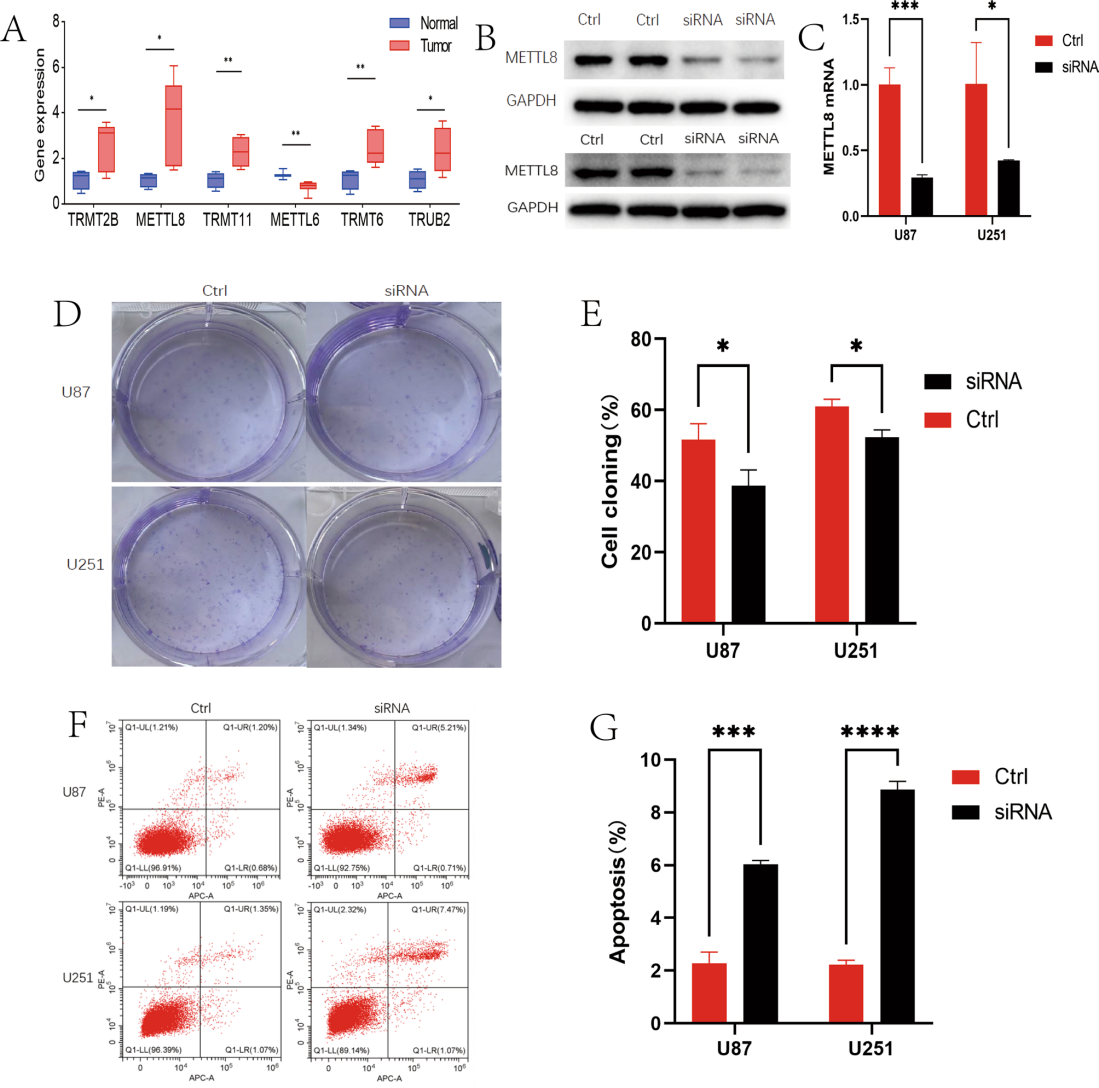
In vitro validation of the role of METTL8 in glioma. (**A**) Validation of 6 MRM-related genes by qPCR in glioma and normal tissues; (**B**) Western blot validated the protein expression of METTL8 in U87 and U251 cells with METTL8 knock-down; (**C**) RT-qPCR revealed the expression of mRNA for METTL8 in U87 and U251 cells with METTL8 knock-down; (**D** and **E**) Cell cloning experiments found that knocking down METTL8 significantly inhibited cell clone formation in U87 and U251 cells; (**F** and **G**) METTL8 knockdown can improve apoptosis of U87 and U251 cells.

To verify some of our hypotheses, we collected 8 glioma tissues including 3 anaplastic astroglioma, 1 anaplastic oligodendroglioma, and 4 astrogliomas and 4 normal tissues for validation of 6 MRM-related genes by qPCR. Results revealed that TRMT2B, TRMT11, METTL8, TRMT6, and TRUB2 were upregulated in glioma, while METTL6 was downregulated (Fig. 11A).

**Figure 12:**
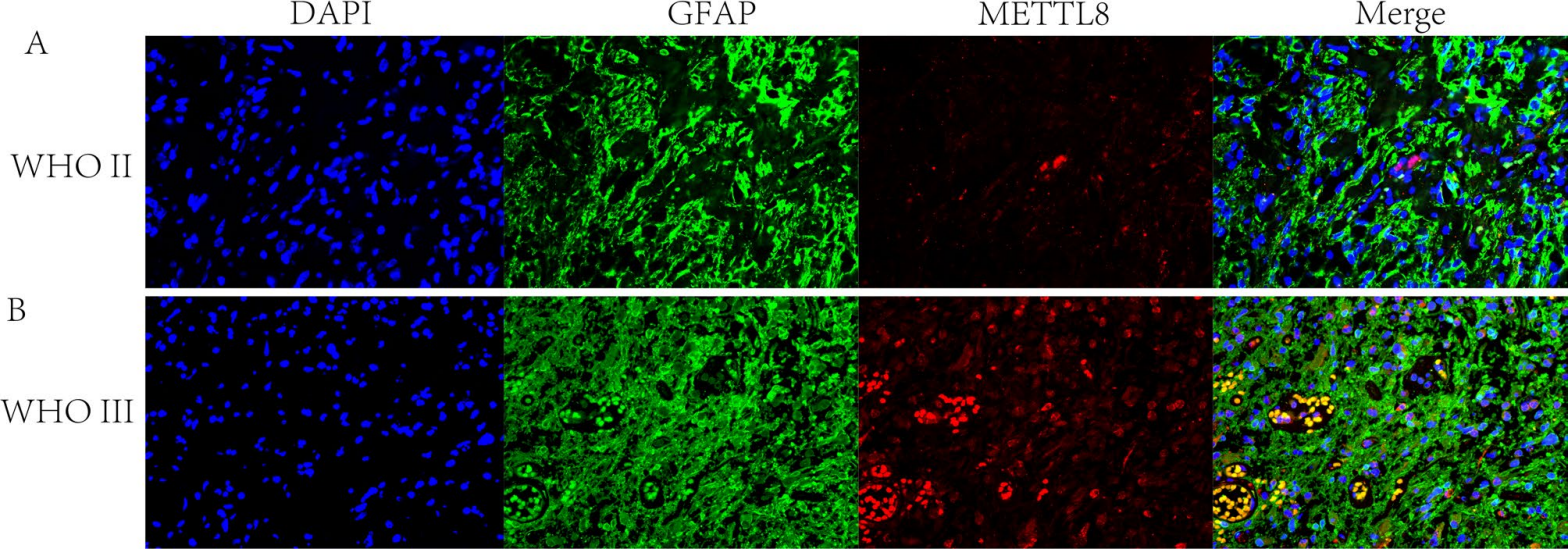
Co-expression of METTL8 and GFAP in LGG tissues. This figure illustrates the expression of METTL8 and GFAP in oligodendroglioma (**A**) and anaplastic astrocytoma (**B**). Cell nuclei were stained with DAPI. GFAP expression is indicated in green, METTL8 expression in red, and their colocalization is depicted in the merged image.

METTL8 was selected to explore its important role in glioma because it has the largest regression coefficient among the genes that make up the MRM signature. During our single-cell data analysis, we discovered that METTL8 was highly expressed in astrocytes. To validate this result, we performed immunofluorescence staining on oligodendroglioma and anaplastic astrocytoma tissue to assess the expression of METTL8 and GFAP. We observed that METTL8 might be involved in the positioning of GFAP (Fig. 12). We then knocked down METTL8 in U87 and U251 (Fig. 11B,C), and clonogenic assay found that knocking down METTL8 significantly inhibited cell colony formation, and the same results were also obtained in U251 cell (Fig. 11D,E). Furthermore, we also assess the effect of METTL8 on apoptosis for glioma. We identified that METTL8 knockdown can promote apoptosis of U87 cell detected by flow cytometry, the result was verified in U251 cell (Fig. 11F,G). All those results indicated that METTL8 was an oncogenic gene of glioma.

## Discussion

Mitochondrial RNAs are modified in a diverse and complex manner in obesity, type 2 diabetes, and cancer^8^. Enhanced reactive oxygen species generation, glycolysis instead of oxidative phosphorylation, and an abnormal mitochondria-mediated apoptotic machinery due to mitochondrial dysfunction are frequently observed in gliomas^34^. A thorough understanding of MRM may shed light on the underlying molecular mechanisms associated with cancers, which remained unclear thus far. Identifying new biomarkers and designing improved treatment strategies for glioma is crucial^8^. We investigated the biological significance of MRM-related genes in LGGs by integrating bulk RNA-seq and scRNA-seq in the present study. The scRNA-seq dataset was retrieved to identify 6 cell types in the LGG microenvironment, and the relationship between MRM-related genes and cell cycle, ICPs, potential BP, and cell–cell communication was assessed. Our discovery indicates that through the NCMA signaling pathway and ICPs, MRM has the ability to control the development of a microenvironment that aids in the advancement of tumors. We conducted bulk RNA-seq analysis in order to comprehensively and systematically assess the expression and prognostic significance of MRM-related genes in LGGs. A superior performance prognostic prediction risk model was established by utilizing MRM-related genes. A nomogram model that predicts the outcome of LGG patients with high accuracy was developed. Patients with high MRM score had high sensitivity to anti-PD1 therapy, AACOCF3, MS.275, AH.6809, tacrolimus, as well as TTNPB.

Thus far, only one study has investigated the biological significance of MRM in cancers^14^. Consequently, the LGG microenvironment lacks extensive discussions regarding the distribution and expression of MRM-related genes throughout the entire cell cycle in various cell types. The bulk RNA-seq analysis for LGGs showed that 22 genes were upregulated, and 10 genes were downregulated compared to normal tissues. Using the TCGA and CGGA datasets, it was discovered that most genes related to MRM were linked to the prognosis of patients with LGG. Previous researches has revealed that MRM-related genes, including METTL8, YRDC, NSUN3, TRMT6, and TRMT61A, were found to be elevated in cancerous tumors and were linked to unfavorable prognostic outcomes^14,35–37^. Furthermore, we conducted scRNA analysis to examine the expression of MRM-related genes across the entire cell cycle in six different cell types. METTL8, METTL2A, TRMT112, and METTL2B were extensively expressed in all cell types. We observed the high expression levels of METTL8, METTL2A, TRMT112, METTL2B, GTPBP3, METTL6, and NSUN4 in cells in the S, G2M, and G1 phases. These results suggest that different cells may have different types of MRM, and future research on MRM in gliomas should consider the cell type.

The association of MRM in the LGG microenvironment was found with the NCAM signaling pathway, which involves the ligand-receptor pairs of NCAM1-NCAM2 and NCAM1-L1CAM, in the present study. Astrocytes, tumor cells, and oligodendrocytes were found to have relatively high gene expression levels within the NCAM signaling pathway network. Astrocytes have been found to have a correlation with the development of glioma^38,39^. For instance, astrocytic differentiation inhibits the progression of glioma^39^. Cellular communication analysis showed that the NCAM signaling network started from astrocytes and oligodendrocytes and targeted other cell types in the glioma microenvironment. Consequently, tumor cells could affect astrocytes and oligodendrocytes via the NCAM signaling pathway. Some regulators of MRM were associated with the expression of NCAM1, NCAM2, and L1CAM, which indicated that MRM may facilitate glioma development via astrocytic modulation. However, the biological mechanism underlying NCAM signaling pathway regulation by MRM remains unknown. In addition, further investigation is needed to confirm whether MRM affects the biological function of astrocytes by regulating the NCAM signaling pathway, leading to the growth of glioma cells.

In bulk RNA-seq analysis, the LASSO and RSF algorithms filtered 6 genes for model construction. Specifically, TRMT6 has been reported to be associated with malignant progression in gliomas. It was discovered that the suppression of cell migration, invasion, and proliferation in gliomas can be achieved by silencing TRMT6^40^. METTL6 and METTL8 are members of the methyltransferase-like gene family^41,42^. METTL6 is essential for the maintenance of stem cell self-renewal and the promotion of hepatoma cell growth^43^. Prior research indicated that METTL8 played a crucial role in the m3c modification of mitochondrial tRNA, thereby enhancing the m3C methylation at position C32 of mt-tRNASer and mt-tRNAThr, and ultimately, guaranteeing optimal composition and function of the mitochondrial respiratory chain^44–48^. However, limited studies have examined the effect of TRMT2B, TRMT11, and TRUB2 in cancers. In the present study, we found that all 6 genes exhibited differential expression between normal tissues and LGGs and were associated with prognosis. In vitro experiments have shown that knocking down METTL8 can significantly inhibit the proliferation and promote apoptosis of glioma cells. METTL8, a methyltransferase acting on RNA, has been revealed to target m3 C32 modification of mt-tRNASer(UCN) and mt-tRNATh involved with the malignant progression of cancers^36,47^. Some evidence indicated that METTL8 was not believed to impact tRNAs; however, it has been proposed to introduce m3C modifications to cellular mRNAs^49^. It is currently unknown whether METTL8 directly serves as a mitochondrial tRNA modifier or a regulatory factor for tRNA modification. Future research should delve into whether METTL8 mediates malignant progression of glioma through tRNA or mRNA m3C modification. Additionally, our cellular communication analysis suggests that some MRM regulators might alter the malignant progression of gliomas by modulating astrocyte signaling pathways. We have also validated the co-expression of METTL8 and GFAP in gliomas, and the results indicate that METTL8 might be involved in the localized expression of GFAP. However, whether METTL8 and GFAP co-localize in tumor cells or astrocytes requires further study. Whether MRM regulators participate in the development and progression of gliomas by altering the phenotype of astrocytes is an important question that deserves in-depth investigation and should attract attention in subsequent related research.

The MRM score based on the 6 genes can be used to differentiate the malignant features of LGGs and classify LGGs into two groups. The MRM score demonstrated high predictive ability for the prognosis and clinical characteristics of patients with gliomas. Patients with high MRM score had a poor clinical outcome, which may be associated with that IDH1 wild-type, high grade tumor, 1p19q non-codeletion, and MGMT promoter nonmethylation were enriched in high MRM score. Furthermore, patients with high MRM score had high tumor mutation burden, high immune infiltration, and high tumor metabolic activity, which may contribute to the shorter survival time of LGG patients. Furthermore, we noted that the low MRM score group exhibited high expression levels of ADORA2A, CX3CL1, EDNRB, HMGB1, IL12A, and VTCN1, while the high MRM score group exhibited high expression levels of all immunomodulators except for VEGFB, TNFSF9, TNF, TLR4, TIGIT, SELP, LAG3, IL13, IL4, IL1B, IFNA2, and IFNA1. In glioma cells, it was observed that the expression of genes related to MRM, like GTPBP3, METTL2A, METTL6, METTL8, NSUN4, and TRMT61A, showed positive correlation with the expression of HAVCR2, PVR, TNFRSF25 and TNFSF15 as revealed by scRNA-seq analysis. A similar correlation between MRM-related genes and ICPs in other cell types was observed. Taken together, the results suggest that MRM may regulate the immune microenvironment of gliomas via immunomodulators. Furthermore, we assessed the capability of the MRM score in predicting the immunotherapy response of patients with LGGs. Patients having a high MRM score showed a high response rate to immunotherapy, presumably due to high immune cell infiltration and ICP expression. The top 5 sensitive drugs (AACOCF3, MS.275, AH.6809, tacrolimus, and TTNPB) identified in this study should be taken into consideration when selecting patients with high MRM score for radical treatment. AACOCF3, an inhibitor of the 85-kDa cytosolic group IV phospholipase A2 (cPLA2), has been showed to effectively suppress ATP production and tumor growth in glioma^50^. MS.275 known as a histone deacetylase inhibitor may act as a potent drug for experimental therapy of glioma^51^. MS.275 may inhibit the offer a new strategy to improve the sensitivity of radiotherapy for glioma via inhibiting histone deacetylase^52^. AH.6809 was a prostaglandin E receptor antagonist and has been suggested to inhibit the growth of glioma cell lines in vitro^53^. Tacrolimus has been widely used as an immunosuppressant for anti-rejection therapy in organ transplantation^54^ and the chemosensitivity to anticancer drugs mediated by tacrolimus also identified by several studies^55^. Although these drugs have been reported to possess antitumor effects, further research might be needed to determine whether they can penetrate the blood–brain barrier and be applied in clinical settings.

Although we evaluated the predictive role of MRM in the LGG microenvironment for the first time, some limitations should be considered. First, we acquired all scRNA-seq and bulk RNA-seq data from public databases rather than using our tumor samples, possibly causing sampling bias. Second, the hypothesis of the current study should be validated with in vivo and in vitro experiments. As a result, we plan to examine the biological effect of MRM in gliomas through mitochondriomics, metabonomics, and transcriptomics in the near future. Third, it was not appropriate to study low-grade gliomas using U87 and U251 cell lines derived from glioblastoma. To address this issue, we will introduce IDH mutations into the U87 and U251 cell lines in future study and further analyze the alterations of MRM genes in these cell lines.

## Conclusion

In summary, single-cell analyses indicated that MRM could play a role in creating a microenvironment conducive to tumor progression through the NCMA signaling pathway and ICPs. A prognostic risk prediction model based on MRM-related genes was developed, demonstrating outstanding performance. Additionally, a nomogram was constructed to predict the prognosis of patients with LGG, which also showed promising results. We further identified two groups of patients with distinct MRM-related gene expression patterns and predicted their responses to ICP inhibitors. The top five most effective drugs for patients with high MRM scores were identified. These novel findings regarding the biological impact of MRM in the glioma microenvironment could facilitate the advancement of targeted therapies and immunotherapies.

### Supplementary Information

Supplementary Figure S1.

Supplementary Figure S2.

Supplementary Figure S3.

Supplementary Figure S4.

Supplementary Figure S5.

Supplementary Figure S6.

Supplementary Figure S7.

Supplementary Figure S8.

Supplementary Figure S9.

Supplementary Figure S10.

Supplementary Legends.

Supplementary Table S1.

Supplementary Table S2.

Supplementary Table S3.

Supplementary Table S4.

## Data Availability

All data are available in a public, open access repository. R, other custom scripts for analyzing data are available upon reasonable request.

## References

[1] Ostrom QT, Gittleman H, Farah P, Ondracek A, Chen Y, Wolinsky Y, Stroup NE, Kruchko C, Barnholtz-Sloan JS (2013). CBTRUS statistical report: Primary brain and central nervous system tumors diagnosed in the United States in 2006–2010. Neuro Oncol..

[2] Cancer Genome Atlas Research Network (2015). Comprehensive, integrative genomic analysis of diffuse lower-grade gliomas. New Engl. J. Med..

[3] Youssef G, Miller JJ (2020). Lower grade gliomas. Curr. Neurol. Neurosci. Rep..

[4] Jiang T, Mao Y, Ma W, Mao Q, You Y, Yang X, Jiang C, Kang C, Li X, Chen L (2016). CGCG clinical practice guidelines for the management of adult diffuse gliomas. Cancer Lett..

[5] Hayes J, Yu Y, Jalbert LE, Mazor T, Jones LE, Wood MD, Walsh KM, Bengtsson H, Hong C, Oberndorfer S (2018). Genomic analysis of the origins and evolution of multicentric diffuse lower-grade gliomas. Neuro Oncol..

[6] Louis DN, Perry A, Wesseling P, Brat DJ, Cree IA, Figarella-Branger D, Hawkins C, Ng HK, Pfister SM, Reifenberger G (2021). The 2021 WHO classification of tumors of the central nervous system: A summary. Neuro Oncol..

[7] Louis DN, Perry A, Reifenberger G, von Deimling A, Figarella-Branger D, Cavenee WK, Ohgaki H, Wiestler OD, Kleihues P, Ellison DW (2016). The 2016 World Health Organization classification of tumors of the central nervous system: A summary. Acta Neuropathol..

[8] Boughanem H, Böttcher Y, Tomé-Carneiro J, de las Hazas ML, Dávalos A, Cayir A, Macias-González M (2022). The emergent role of mitochondrial RNA modifications in metabolic alterations. WIREs RNA.

[9] Pan T (2018). Modifications and functional genomics of human transfer RNA. Cell Res..

[10] Suzuki T, Yashiro Y, Kikuchi I, Ishigami Y, Saito H, Matsuzawa I, Okada S, Mito M, Iwasaki S, Ma D (2020). Complete chemical structures of human mitochondrial tRNAs. Nat. Commun..

[11] Suzuki T (2021). The expanding world of tRNA modifications and their disease relevance. Nat. Rev. Mol. Cell Biol..

[12] Asano K, Suzuki T, Saito A, Wei F-Y, Ikeuchi Y, Numata T, Tanaka R, Yamane Y, Yamamoto T, Goto T (2018). Metabolic and chemical regulation of tRNA modification associated with taurine deficiency and human disease. Nucleic Acids Res..

[13] O’Sullivan M, Rutland P, Lucas D, Ashton E, Hendricks S, Rahman S, Bitner-Glindzicz M (2015). Mitochondrial m. 1584A 12S m62A rRNA methylation in families with m. 1555A> G associated hearing loss. Human Mol. Genet..

[14] Delaunay S, Pascual G, Feng B, Klann K, Behm M, Hotz-Wagenblatt A, Richter K, Zaoui K, Herpel E, Münch C (2022). Mitochondrial RNA modifications shape metabolic plasticity in metastasis. Nature.

[15] Lonsdale J, Thomas J, Salvatore M, Phillips R, Lo E, Shad S, Hasz R, Walters G, Garcia F, Young N (2013). The Genotype-Tissue Expression (GTEx) project. Nat Genet.

[16] Hao Y, Hao S, Andersen-Nissen E, Mauck WM, Zheng S, Butler A, Lee MJ, Wilk AJ, Darby C, Zager M (2021). Integrated analysis of multimodal single-cell data. Cell.

[17] Zhang X, Lan Y, Xu J, Quan F, Zhao E, Deng C, Luo T, Xu L, Liao G, Yan M (2019). Cell Marker: A manually curated resource of cell markers in human and mouse. Nucleic Acids Res..

[18] Hänzelmann S, Castelo R, Guinney J (2013). GSVA: Gene set variation analysis for microarray and RNA-Seq data. BMC Bioinform..

[19] Doncheva NT, Morris JH, Holze H, Kirsch R, Nastou KC, Cuesta-Astroz Y, Rattei T, Szklarczyk D, von Mering C, Jensen LJ (2022). Cytoscape stringApp 2.0: Analysis and visualization of heterogeneous biological networks. J. Proteome Res..

[20] Seiler M, Huang CC, Szalma S, Bhanot G (2010). ConsensusCluster: A software tool for unsupervised cluster discovery in numerical data. OMICS.

[21] Taylor JMG (2011). Random survival forests. J. Thoracic Oncol..

[22] Feng J-W, Ye J, Qi G-F, Hong L-Z, Wang F, Liu S-Y, Jiang Y (2022). LASSO-based machine learning models for the prediction of central lymph node metastasis in clinically negative patients with papillary thyroid carcinoma. Front. Endocrinol. (Lausanne).

[23] Ishwaran H, Lu M (2019). Standard errors and confidence intervals for variable importance in random forest regression, classification, and survival. Stat. Med..

[24] Xiao B, Liu L, Li A, Xiang C, Wang P, Li H, Xiao T (2020). Identification and verification of immune-related gene prognostic signature based on ssGSEA for osteosarcoma. Front. Oncol..

[25] Becht E, Giraldo NA, Lacroix L, Buttard B, Elarouci N, Petitprez F, Selves J, Laurent-Puig P, Sautès-Fridman C, Fridman WH (2016). Estimating the population abundance of tissue-infiltrating immune and stromal cell populations using gene expression. Genome Biol..

[26] Li T, Fan J, Wang B, Traugh N, Chen Q, Liu JS, Li B, Liu XS (2017). TIMER: A web server for comprehensive analysis of tumor-infiltrating immune cells. Cancer Res..

[27] Yoshihara K, Shahmoradgoli M, Martínez E, Vegesna R, Kim H, Torres-Garcia W, Treviño V, Shen H, Laird PW, Levine DA (2013). Inferring tumour purity and stromal and immune cell admixture from expression data. Nat. Commun..

[28] Thorsson V, Gibbs DL, Brown SD, Wolf D, Bortone DS, Ou Yang T-H, Porta-Pardo E, Gao GF, Plaisier CL, Eddy JA (2019). The immune landscape of cancer. Immunity.

[29] Lu X, Jiang L, Zhang L, Zhu Y, Hu W, Wang J, Ruan X, Xu Z, Meng X, Gao J (2019). Immune signature-based subtypes of cervical squamous cell carcinoma tightly associated with human papillomavirus type 16 expression, molecular features, and clinical outcome. Neoplasia.

[30] McGranahan N, Furness AJS, Rosenthal R, Ramskov S, Lyngaa R, Saini SK, Jamal-Hanjani M, Wilson GA, Birkbak NJ, Hiley CT (2016). Clonal neoantigens elicit T cell immunoreactivity and sensitivity to immune checkpoint blockade. Science (1979).

[31] Hoshida Y, Brunet J-P, Tamayo P, Golub TR, Mesirov JP (2007). Subclass mapping: Identifying common subtypes in independent disease data sets. PLoS ONE.

[32] Yang C, Zhang H, Chen M, Wang S, Qian R, Zhang L, Huang X, Wang J, Liu Z, Qin W (2022). A survey of optimal strategy for signature-based drug repositioning and an application to liver cancer. Elife.

[33] Vicario N, Spitale FM, Tibullo D, Giallongo C, Amorini AM, Scandura G, Spoto G, Saab MW, D’Aprile S, Alberghina C (2021). Clobetasol promotes neuromuscular plasticity in mice after motoneuronal loss via sonic hedgehog signaling, immunomodulation and metabolic rebalancing. Cell Death Dis..

[34] Guntuku L, Naidu VGM, Ganesh YV (2016). Mitochondrial dysfunction in gliomas: Pharmacotherapeutic potential of natural compounds. Curr. Neuropharmacol..

[35] Su Z, Monshaugen I, Wilson B, Wang F, Klungland A, Ougland R, Dutta A (2022). TRMT6/61A-dependent base methylation of tRNA-derived fragments regulates gene-silencing activity and the unfolded protein response in bladder cancer. Nat. Commun..

[36] Schöller E, Marks J, Marchand V, Bruckmann A, Powell CA, Reichold M, Mutti CD, Dettmer K, Feederle R, Hüttelmaier S (2021). Balancing of mitochondrial translation through METTL8-mediated m3C modification of mitochondrial tRNAs. Mol. Cell.

[37] Shen H, Zheng E, Yang Z, Yang M, Xu X, Zhou Y, Ni J, Li R, Zhao G (2020). YRDC is upregulated in non-small cell lung cancer and promotes cell proliferation by decreasing cell apoptosis. Oncol. Lett..

[38] Adhikari AS, Sullivan T, Bargaje R, Lu L, O’Sullivan TN, Song Y, van Dyke T (2022). Abrogation of Rb tumor suppression initiates GBM in differentiated astrocytes by driving a progenitor cell program. Front. Oncol..

[39] Trovato F, Stefani FR, Li J, Zetterdahl OG, Canals I, Ahlenius H, Bengzon J (2022). Transcription factor forced astrocytic differentiation impairs human glioblastoma growth in vitro and in vivo. Mol. Cancer Ther..

[40] Wang B, Niu L, Wang Z, Zhao Z (2021). RNA m1A methyltransferase TRMT6 predicts poorer prognosis and promotes malignant behavior in glioma. Front. Mol. Biosci..

[41] Wong JM, Eirin-Lopez JM (2021). Evolution of methyltransferase-like (METTL) proteins in metazoa: A complex gene family involved in epitranscriptomic regulation and other epigenetic processes. Mol. Biol. Evol..

[42] Tooley JG, Catlin JP, Tooley CES (2022). METTLing in stem cell and cancer biology. Stem Cell Rev. Rep..

[43] Ignatova VV, Kaiser S, Ho JSY, Bing X, Stolz P, Tan YX, Lee CL, Gay FPH, Lastres PR, Gerlini R (2020). METTL6 is a tRNA m3 methyltransferase that regulates pluripotency and tumor cell growth. Sci. Adv..

[44] Lentini JM, Bargabos R, Chen C, Fu D (2022). Methyltransferase METTL8 is required for 3-methylcytosine modification in human mitochondrial tRNAs. J. Biol. Chem..

[45] Huang M-H, Peng G-X, Mao X-L, Wang J-T, Zhou J-B, Zhang J-H, Chen M, Wang E-D, Zhou X-L (2022). Molecular basis for human mitochondrial tRNA m3C modification by alternatively spliced METTL8. Nucleic Acids Res..

[46] Kowalinski E, Alfonzo JD (2021). METTLing in the right place: METTL8 is a mitochondrial tRNA-specific methyltransferase. Mol. Cell.

[47] Zhang L-H, Zhang X-Y, Hu T, Chen X-Y, Li J-J, Raida M, Sun N, Luo Y, Gao X (2020). The SUMOylated METTL8 induces R-loop and tumorigenesis via m3C. iScience.

[48] Kleiber N, Lemus-Diaz N, Stiller C, Heinrichs M, Mai MM-Q, Hackert P, Richter-Dennerlein R, Höbartner C, Bohnsack KE, Bohnsack MT (2022). The RNA methyltransferase METTL8 installs m3C32 in mitochondrial tRNAsThr/Ser(UCN) to optimise tRNA structure and mitochondrial translation. Nat. Commun..

[49] Xu L, Liu X, Sheng N, Oo KS, Liang J, Chionh YH, Xu J, Ye F, Gao Y-G, Dedon PC (2017). Three distinct 3-methylcytidine (m3C) methyltransferases modify tRNA and mRNA in mice and humans. J. Biol. Chem..

[50] Yang S, Zhao J, Cui X, Zhan Q, Yi K, Wang Q, Xiao M, Tan Y, Hong B, Fang C (2022). TCA-phospholipid-glycolysis targeted triple therapy effectively suppresses ATP production and tumor growth in glioblastoma. Theranostics.

[51] Eyupoglu IY, Hahnen E, Trankle C, Savaskan NE, Siebzehnrubl FA, Buslei R, Lemke D, Wick W, Fahlbusch R, Blumcke I (2006). Experimental therapy of malignant gliomas using the inhibitor of histone deacetylase MS-275. Mol. Cancer Ther..

[52] Essien EI, Hofer TP, Atkinson MJ, Anastasov N (2022). Combining HDAC and MEK inhibitors with radiation against glioblastoma-derived spheres. Cells.

[53] Matsuo M, Yoshida N, Zaitsu M, Ishii K, Hamasaki Y (2004). Inhibition of human glioma cell growth by a PHS-2 inhibitor, NS398, and a prostaglandin E receptor subtype EP1-selective antagonist, SC51089. J. Neurooncol..

[54] Lao Q, Wu X, Zheng X, Hu J, Huang S, Li D, Du Y, Yang N, Zhu H (2024). Effect of tacrolimus time in therapeutic range on postoperative recurrence in patients undergoing liver transplantation for liver cancer. Ther. Drug Monit..

[55] Garrido W, Muñoz M, San Martín R, Quezada C (2011). FK506 confers chemosensitivity to anticancer drugs in glioblastoma multiforme cells by decreasing the expression of the multiple resistance-associated protein-1. Biochem. Biophys. Res. Commun..

