# Supplementary figures and images for: Mitochondrial RNA modification-based signature to predict prognosis of lower grade glioma: a multi-omics exploration and verification study
Source: Sci Rep. 2024 Jun 1;14:12602. doi: 10.1038/s41598-024-63592-w (PMC11144219; doi:10.1038/s41598-024-63592-w)

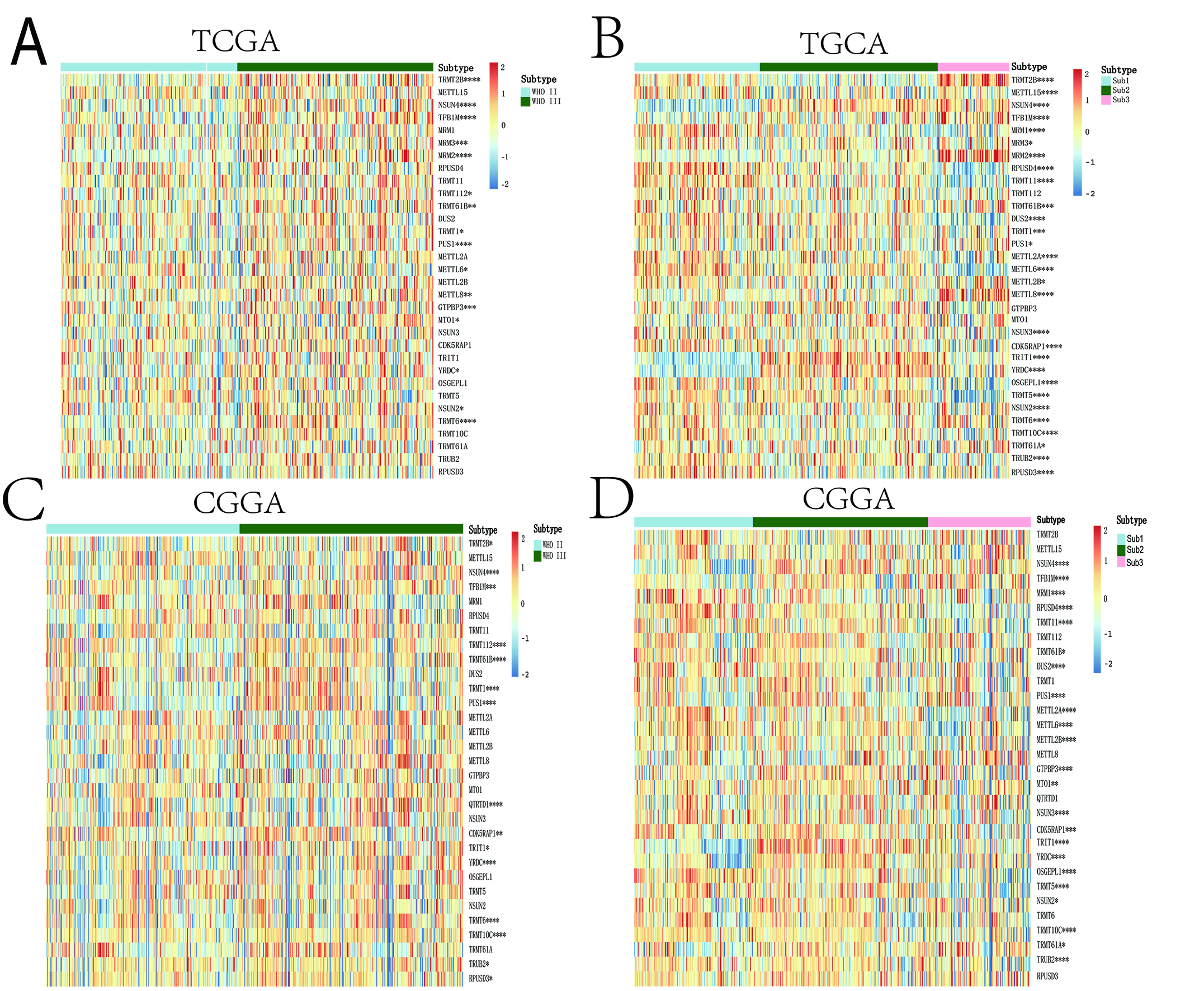

Supplement: Supplementary file 1 — Supplementary Figure S1. [file 41598_2024_63592_MOESM1_ESM.tif]

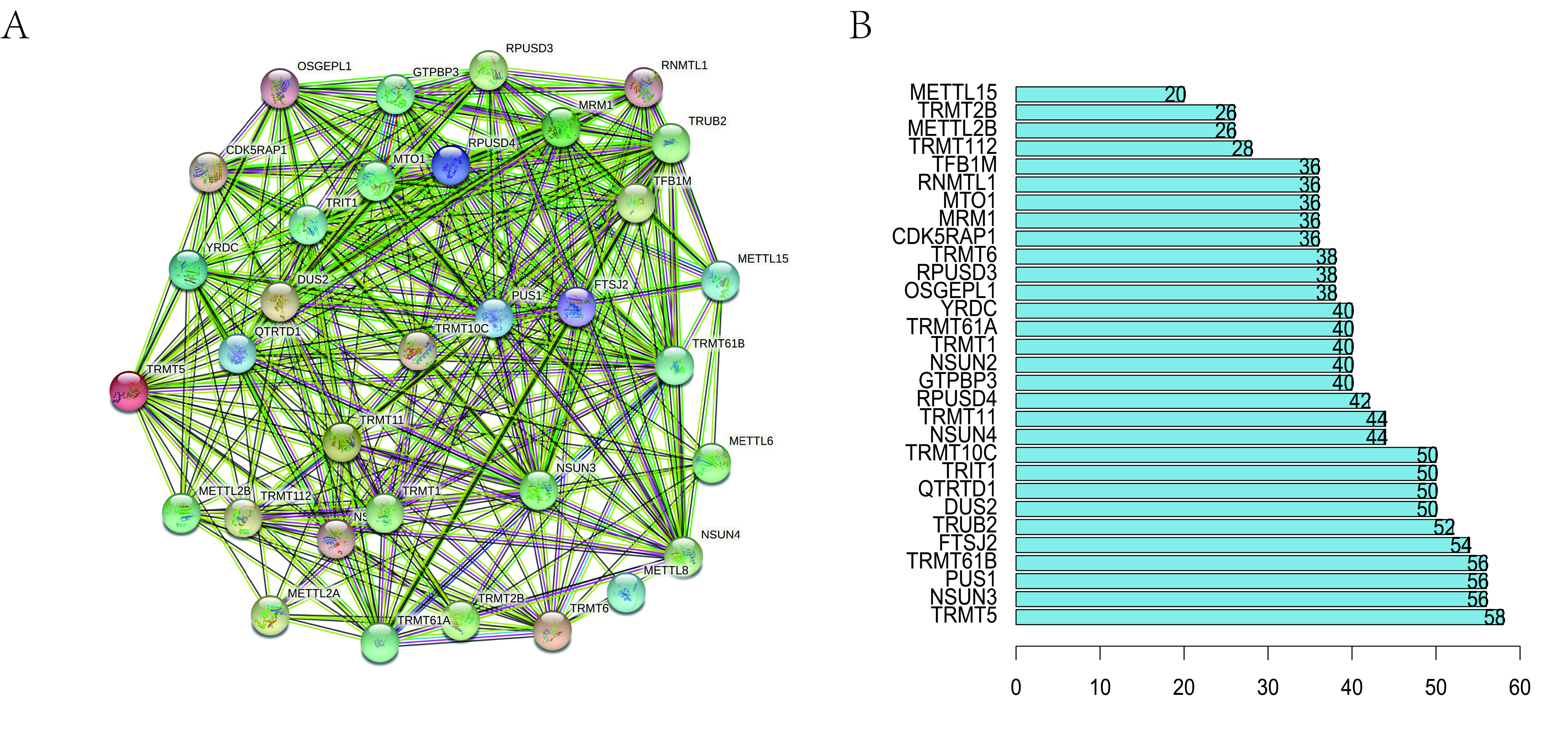

Supplement: Supplementary file 2 — Supplementary Figure S2. [file 41598_2024_63592_MOESM2_ESM.tif]

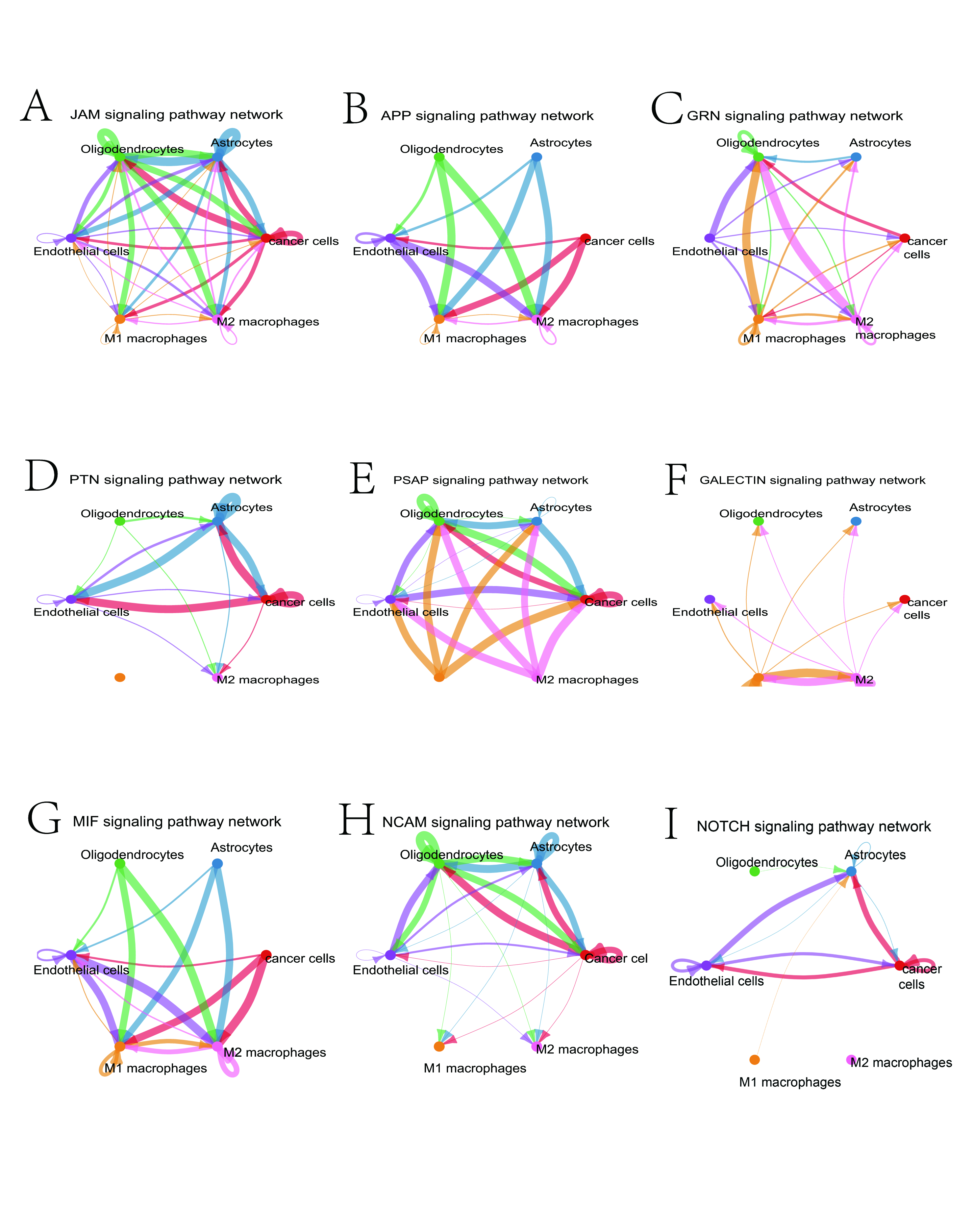

Supplement: Supplementary file 5 — Supplementary Figure S5. [file 41598_2024_63592_MOESM5_ESM.tif]

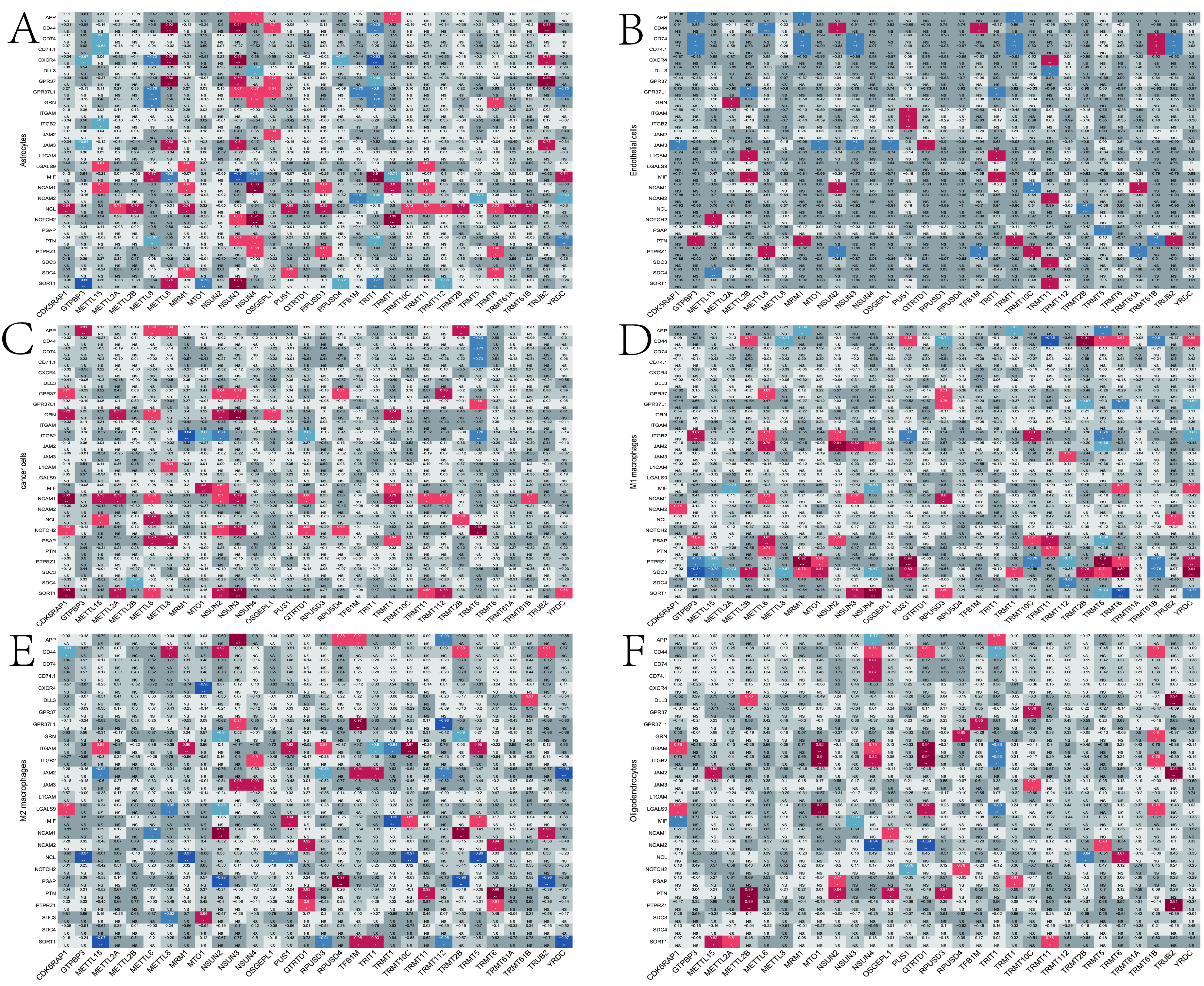

Supplement: Supplementary file 6 — Supplementary Figure S6. [file 41598_2024_63592_MOESM6_ESM.tif]

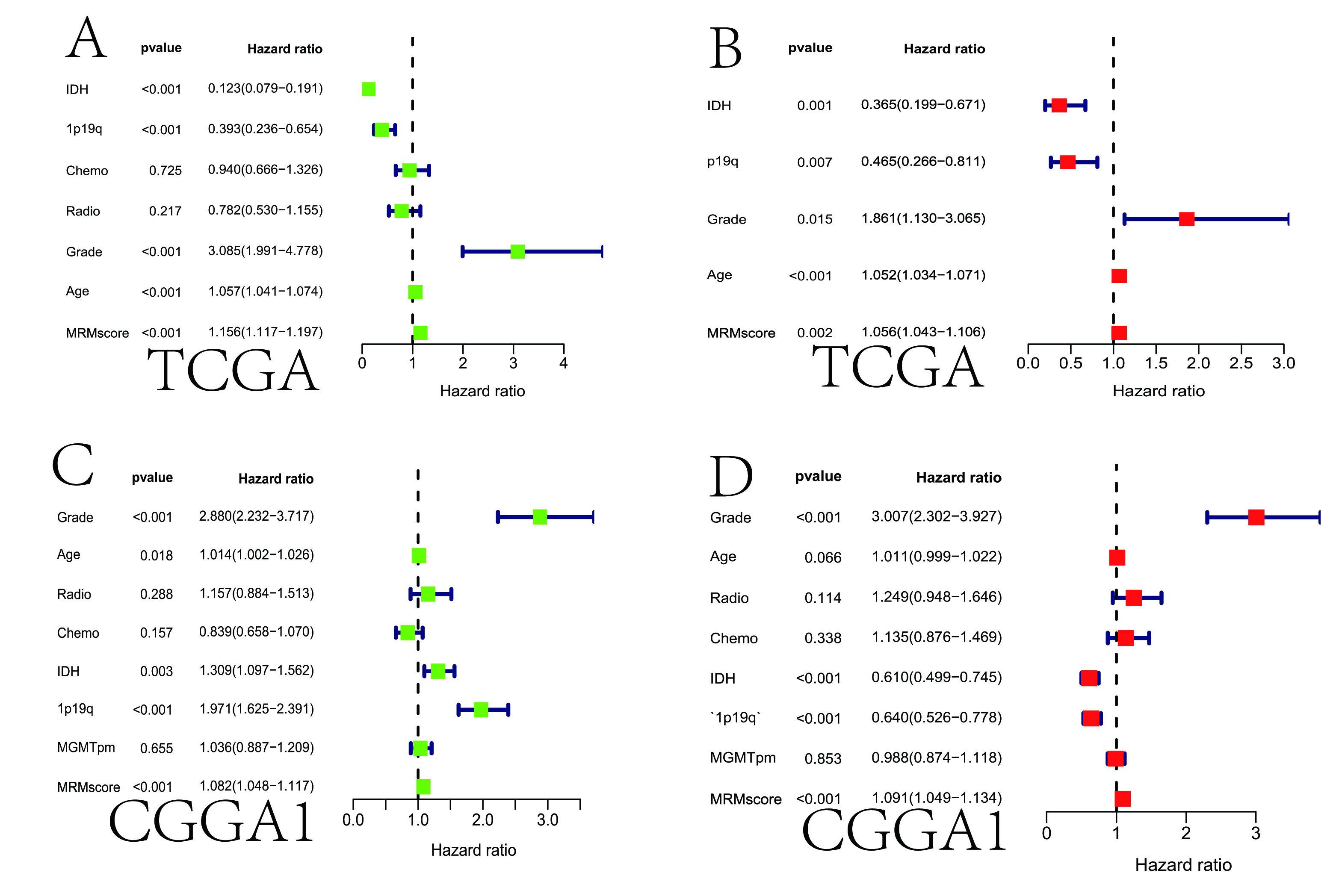

Supplement: Supplementary file 7 — Supplementary Figure S7. [file 41598_2024_63592_MOESM7_ESM.tif]

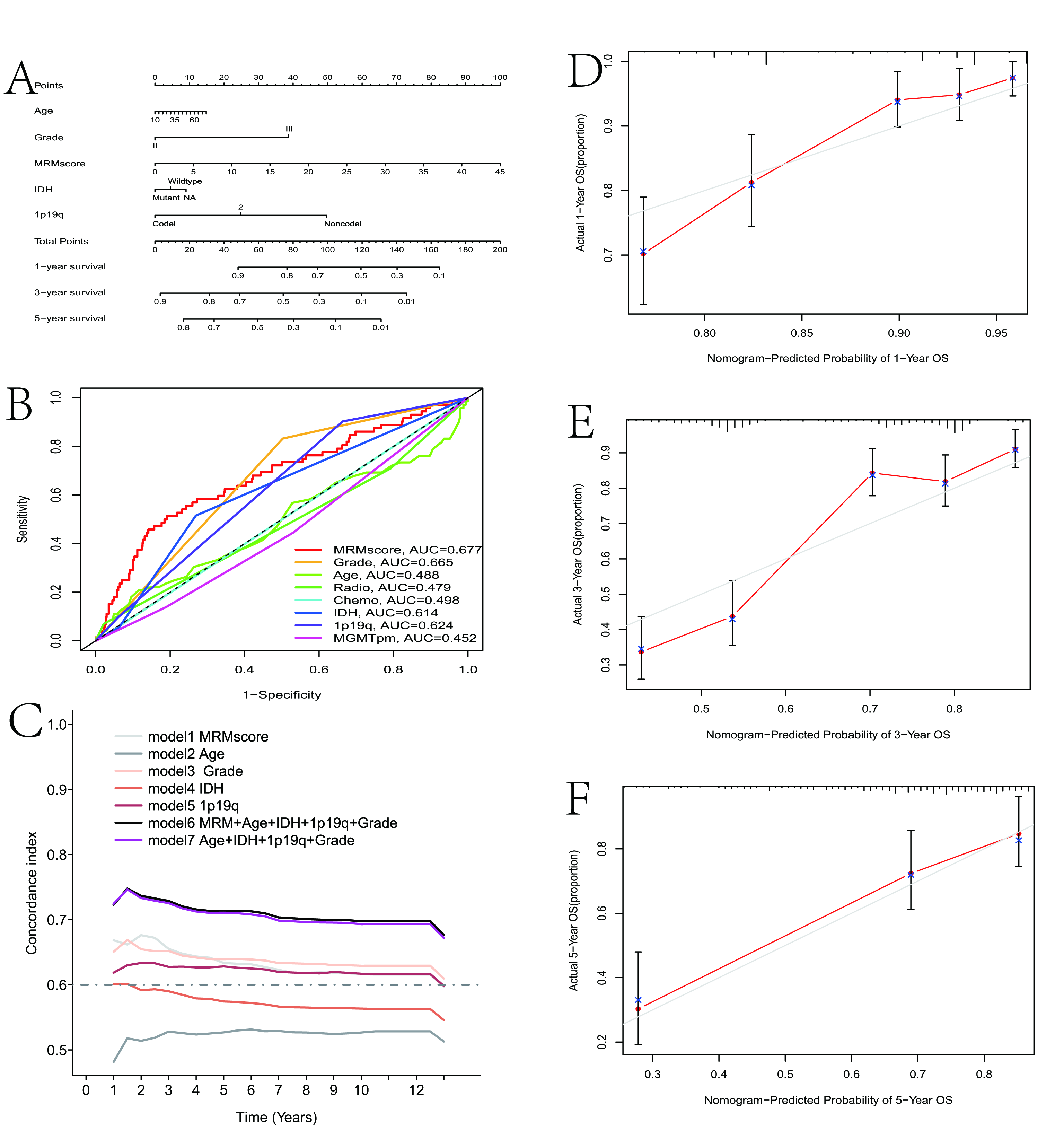

Supplement: Supplementary file 8 — Supplementary Figure S8. [file 41598_2024_63592_MOESM8_ESM.tif]
