# Supplementary material for: Mitochondrial RNA modification-based signature to predict prognosis of lower grade glioma: a multi-omics exploration and verification study
Source: Sci Rep. 2024 Jun 1;14:12602. doi: 10.1038/s41598-024-63592-w (PMC11144219; doi:10.1038/s41598-024-63592-w)
Supplement: Supplementary file 11 — Supplementary Legends. [file 41598_2024_63592_MOESM11_ESM.docx]

**Mitochondrial RNA modification-based signature to predict prognosis of lower grade glioma: A Multi-Omics Exploration and Verification Study**

Figure S1:The functional modules of MRM related signature. PPI network of sub1(A), sub2(B) and sub3(C) in astrocyte; PPI network of sub1(D), sub2(E) and sub3(F) in endothelial cell; PPI network of sub1(G), sub2(H) and sub3(I) in glioma cell; PPI network of sub1(J), sub2(K) and sub3(L) in M1 macrophages; PPI network of sub1(M), sub2(N) and sub3(O) in M2 macrophages; PPI network of sub1(P), sub2(Q) and sub3(R) in oligodendrocytes.

Figure S2: Cell communication analysis of LGG microenvironment identified the robust signaling pathway.

Figure S3: Correlation analysis of MRM-related genes in signaling pathway networks in 6 cell types. (A) astrocyte; (B) endothelial cell; (C) glioma cell; (D) M1 macrophages; (E) M2 macrophages; (F) oligodendrocytes.

Figure S4:The expression of MRM-related genes among different tumor grade and subtypes based on IDH1 mutation and 1p19q codel in TCGA (A and B) and CGGA1 (C and D) datasets.

Figure S5:Analysis of the interaction patterns among the 35 MRM-related genes. A: PPI of MRM-related genes; B: TRMT5 was the hub node of MRM-related genes.

Figure S6: Genes with mutation rate greater than 10% associated with MRM-related genes.

Figure S7: Univariate and multivariate analysis for effect of MRMscore. (A) univariate analysis in TCGA database; (B) multivariate analysis in TCGA database; (C) univariate analysis in CGGA1 database; (D) multivariate analysis in CGGA1 database.

Figure S8: Prognostic nomogram based on the risk model in CGGA1 database. (A) the nomogram based on the risk model; (B) the ROC curve reveal that the risk model has a higher area under the curve (AUC) than the factors of age, IDH mutation status, 1p19q codeletion status, and tumor grade; (C) the concordance index of model based on MRM score and parameters; (D-F) The calibration curve of 1-year, 3-year and 5-year OS based on the nomogram, respectively.

Figure S9: The differentially expressed genes, functional annotation, and metabolism between low-MRM score and high-MRM score group. A and B: the differentially expressed genes among MRM group; C: GO analysis; D: KEEG analysis; E: differential metabolism between low- and high-MRM score group.

Figure S10: Immune infiltration for MRM signature in CGGA1 cohort. (A) infiltration of immune cells among MRM signature groups; distribution of ESTIMATEscore(B), immunescore(C), stromalscore(D), and tumor purity(E) among MRM signature subgroups.

Table S1-S4: Table S1 Primer pairs of 6 MRM-related genes. Table S2 Summary of mitochondrial modification related genes; Table S3 MRM related gene clusters in 6 cell types and functional annotation; Table S4 Results of cellular communication analysis.
